# Quantitative super-resolution imaging of Bruchpilot distinguishes active zone states

**DOI:** 10.1038/ncomms5650

**Published:** 2014-08-18

**Authors:** Nadine Ehmann, Sebastian van de Linde, Amit Alon, Dmitrij Ljaschenko, Xi Zhen Keung, Thorge Holm, Annika Rings, Aaron DiAntonio, Stefan Hallermann, Uri Ashery, Manfred Heckmann, Markus Sauer, Robert J. Kittel

**Affiliations:** 1 Department of Neurophysiology, Institute of Physiology, University of Würzburg, 97070 Würzburg, Germany; 2 Department of Biotechnology and Biophysics, University of Würzburg, 97074 Würzburg, Germany; 3 Department of Neurobiology, Wise Faculty of Life Sciences, Tel Aviv University, Tel Aviv 69978, Israel; 4 European Neuroscience Institute, University Medical Center Göttingen, 37077 Göttingen, Germany; 5 Carl-Ludwig-Institute of Physiology, Medical Faculty, University of Leipzig, 04103 Leipzig, Germany; 6 Department of Developmental Biology, Washington University School of Medicine, St. Louis, Missouri 63110, USA; 7 Sagol School of Neuroscience, Tel Aviv University, Tel Aviv 69978, Israel; 8 These authors contributed equally to this work

## Abstract

The precise molecular architecture of synaptic active zones (AZs) gives rise to different structural and functional AZ states that fundamentally shape chemical neurotransmission. However, elucidating the nanoscopic protein arrangement at AZs is impeded by the diffraction-limited resolution of conventional light microscopy. Here we introduce new approaches to quantify endogenous protein organization at single-molecule resolution *in situ* with super-resolution imaging by direct stochastic optical reconstruction microscopy (*d*STORM). Focusing on the *Drosophila* neuromuscular junction (NMJ), we find that the AZ cytomatrix (CAZ) is composed of units containing ~137 Bruchpilot (Brp) proteins, three quarters of which are organized into about 15 heptameric clusters. We test for a quantitative relationship between CAZ ultrastructure and neurotransmitter release properties by engaging *Drosophila* mutants and electrophysiology. Our results indicate that the precise nanoscopic organization of Brp distinguishes different physiological AZ states and link functional diversification to a heretofore unrecognized neuronal gradient of the CAZ ultrastructure.

## Abstract (web summary)

Complex molecular interactions occur in the active zone cytomatrix (CAZ) within the presynaptic terminal to regulate synaptic plasticity. Here, the authors use imaging techniques to show that the CAZ is composed of units containing on average 137 Bruchpilot proteins, many of which are arranged into clusters.

## Graphical abstract

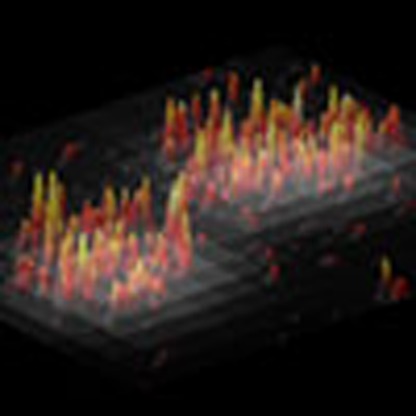

A major challenge facing the scientific exploration of brain function is the accurate interpretation of structure–function relationships^1^. The synaptic active zone (AZ) is a specialization within the presynaptic terminal, which is functionally defined as the site of neurotransmitter release and can be morphologically identified by the proteinaceous AZ cytomatrix (CAZ)^2^. At AZs, complex molecular interactions deliver the speed, precision and plasticity unique to neurotransmission^3^. The CAZ is believed to provide a structural platform for these interactions. In electron microscopy (EM), the CAZ is revealed as an electron-dense structure, whose morphology varies between different types of neurons and between individual synapses formed by the same partner cells. Importantly, activity-dependent structural variations of the CAZ can occur at individual synapses in a dynamic manner and appear to correlate with functional properties of transmitter release^4^,^5^,^6^,^7^. The mechanistic coupling of molecular composition, CAZ structure and neurotransmission, however, remains largely elusive.

Despite a gradually emerging comprehensive protein catalogue, we still lack basic information describing how the nanoscopic organization of proteins gives rise to synaptic function. In essence, this is because of the diffraction-limited resolution of light microscopy that has hindered access to the spatial nanodomain in a physiologically relevant context. In recent years, super-resolution imaging methods have emerged that enable far-field fluorescence microscopy with spatial resolutions approaching virtually EM^8^,^9^,^10^,^11^,^12^,^13^. Here, super-resolution imaging by single-molecule photoactivation or photoswitching and position determination (localization microscopy) captures an outstanding position because it reconstructs the super-resolved image from single-molecule localization events. Thus, it can deliver information about molecular distributions, even giving absolute number of proteins present in subcellular compartments, provided that each molecule is labelled with an intact fluorophore, detected above a certain photon threshold, and that the number of localizations measured per individual fluorophore is accessible^14^. This provides insight into biological systems at a molecular level.

Neurotransmitter release is controlled by a multi-step process of vesicle delivery and molecular maturation at the AZ to generate fusion-competent synaptic vesicles. The availability of such readily releasable vesicles (RRVs) and their calcium-dependent probability of fusion fundamentally determine synaptic performance^15^. The precise spatial organization of AZ constituents shapes such basic elements of presynaptic function and contributes to use-dependent synaptic plasticity. Here, we set out to test whether a quantitative analysis of the CAZ ultrastructure could provide information on these functional properties.

To this end, we focused specifically on Bruchpilot (Brp), a major structural and functional component of the CAZ in *Drosophila*. Brp performs a dual function of clustering Ca^2+^ channels and concentrating synaptic vesicles at neuromuscular AZs^16^,^17^. By promoting excitation–secretion coupling, Brp supports efficient transmitter release, shapes synaptic plasticity^5^,^18^ and participates in certain forms of learning^19^. Using STED (stimulated emission depletion) microscopy, previous work has provided an ultrastructural description of the orientation of Brp in the CAZ^16^,^20^,^21^. Building upon this basic understanding, we sought to extract quantitative data on the number and precise spatial arrangement of Brp molecules within AZs.

The present study puts forward a novel approach to extract protein counts from macromolecular assemblies at single-molecule resolution. We demonstrate this procedure by estimating endogenous Brp protein copies in their native environment by direct stochastic optical reconstruction microscopy (*d*STORM)^11^,^22^. *Drosophila* mutants were used to analyse different AZ states, and electrophysiology was applied to functionally calibrate super-resolution images. Our results demonstrate that functional information on neurotransmission is provided by the nanoscopic organization of Brp.

## Results

### Localization microscopy of the CAZ nanostructure

**Figure 1 f1:**
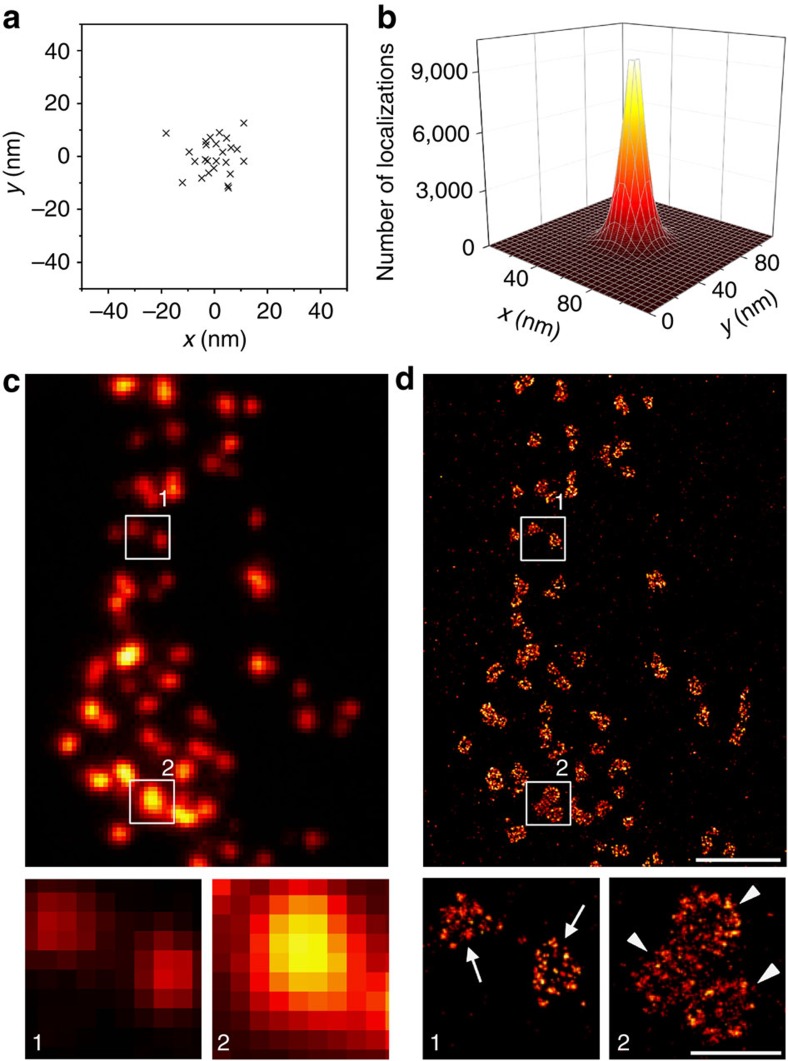
*d*STORM resolves substructural information on the CAZ. (**a**) 2D localization pattern of a single, unspecifically bound Cy5 F(ab′)_2_ fragment. (**b**) Aligned distribution of 209,537 localizations from 21,436 unspecifically bound antibodies. A 2D Gaussian fit gives a localization precision (s.d.) of 7.16±0.02 nm. (**c**,**d**) *d*STORM (right panels) uncovers a substructural organization of the CAZ, disguised in epifluorescence images (left panels) of wt NMJs stained against Brp. Thereby, assemblies of Brp into clusters, termed CAZ-units, could be identified. Panel **d**1 shows two separate AZs (arrows), viewed *en face*, each containing one CAZ-unit, and panel **d**2 shows a single AZ composed of three CAZ-units (arrowheads). Lower panels display magnification of boxed regions. Scale bars, 2 μm (**c**,**d**) and 500 nm (**c**1,**c**2,**d**1,**d**2).

To obtain detailed structural information on the CAZ *in situ*, we used *d*STORM at the glutamatergic larval *Drosophila* neuromuscular junction (NMJ). The CAZ was recognized with a monoclonal antibody (mAb Brp^Nc82^) directed against a C-terminal epitope of Brp^20^,^23^. To optimize structural resolution, we used a secondary F(ab′)_2_ fragment labelled on average with 1.3 Cy5 fluorophores. With an IgG size of 8–10 and ~4 nm for the F(ab′)_2_ fragment, we estimate ~13 nm for the spatial dimensions of the antibody–fluorophore complex^24^,^25^. Localization microscopy relies on temporally separating fluorescence emission from single fluorophores within a diffraction-limited area. The position of single-molecule fluorescence signals can then be precisely determined (localized) by fitting of a two-dimensional (2D) Gaussian function to the point-spread function. The localizations of all emitters are finally used to reconstruct a super-resolution image^10^,^11^,^12^,^13^. Owing to the high brightness of Cy5, a localization precision of 6–7 nm was achieved (s.d., in lateral direction) using either localizations of individual, isolated fluorophore-labelled antibodies in the sample or nearest neighbour analysis^26^ (Fig. 1a,b and Supplementary Fig. 1; see Methods section).

At roughly 200 kDa, Brp is a large coiled-coil domain protein that adopts an elongated and polarized orientation perpendicular to the AZ membrane. Its membrane-proximal N terminus helps to cluster Ca^2+^ channels at the AZ, whereas the C-terminal region of Brp reaches into the cytoplasm to tether synaptic vesicles^16^,^17^,^20^. Thus, localizations defined by mAb Brp^Nc82^ correspond to the distal region of macromolecular CAZ-filaments observed in electron micrographs of high-pressure frozen NMJs^20^,^27^. The increase in spatial resolution by *d*STORM uncovered detailed information of the CAZ, concealed in diffraction-limited conventional fluorescence microscopy (Fig. 1c,d). In the present study, we define an individual AZ via its CAZ, which is an interconnected region of Brp immunoreactivity. Within one such Brp assembly, localization microscopy resolved a substructural arrangement of Brp into modules (Fig. 1d). Hereafter, this further level of organization is termed a CAZ-unit. An AZ could contain single, individual (Fig. 1d1, [Fig f1]) or multiple, closely grouped CAZ-units (Fig. 1d2). The same organizational principle was also observed with Alexa Fluor 700 (A700) and Alexa Fluor 532 (A532) fluorophores, although at lower spatial resolution than obtained with Cy5 (Supplementary Fig. 1). For the following analyses, we therefore used Cy5 and considered only single, clearly distinguishable CAZ-units (Fig. 1d1, [Fig f1]).

A wild-type (wt) CAZ-unit (*en face* view, that is, optical axis perpendicular to the membrane) contains on average 1,021±43 (s.e.m.) localizations and, considering its size (0.095±0.003 μm^2^ s.e.m., *n*=144 CAZ-units), is likely analogous to a CAZ structure as defined by STED (termed donut)^16^ and EM (termed T-bar)^28^. Several T-bars may reside at one synapse, and this is reflected by groups of narrowly spaced CAZ-units (for example, triple T-bar AZ in Fig. 1d2). As an AZ consists of 1,257±89 localizations (s.e.m.), it will contain on average 1.2 CAZ-units. This calculation is consistent with the number of T-bars detected per synapse in electron micrographs^28^,^29^.

**Figure 2:**
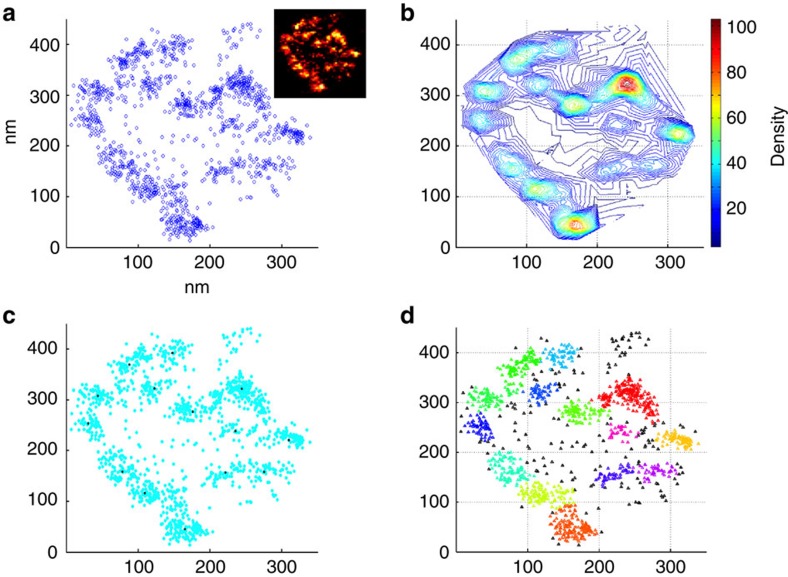
Density-based analysis reveals that CAZ-units are built from multiple Brp clusters. (**a**) Spatial distribution of localizations in a specific CAZ-unit (shown as inset). (**b**) Density distribution of localizations in a 20-nm search radius (number of localizations within Eps-environment colour coded). Difference between elevation lines, 2 nm. (**c**) Centres of mass, that is, local density maxima as discovered by algorithm. (**d**) Final clusters as defined by algorithm. Each colour represents a different cluster. Parameters used for algorithm: 20 nm search radius (Eps) and 16 neighbours threshold (*k*).

Moving on to the next level of organization, we studied the substructure of CAZ-units. EM tomography at the *Drosophila* NMJ has reported macromolecular CAZ-filaments with a diameter of ~10 nm (ref. 27). How many Brp proteins contribute to one filament is, however, unknown. In order to describe the spatial arrangement of Brp molecules in quantitative terms, we analysed the distribution of localizations using a clustering algorithm that was modified from Bar-On *et al*.^30^ and took into account the filament diameter in electron micrographs (Fig. 2 and Supplementary Movie 1; see Methods section). The density-based analysis calculated that an average CAZ-unit contains 14.5±0.4 multiprotein clusters (s.e.m.) with an elliptic shape (29.8±0.2 nm s.e.m. long radius × 19.9±0.1 nm s.e.m. short radius) and an average size of 1,568±21 nm^2^ (s.e.m.). Considering the dimensions of the antibody complex (~13 nm) and our localization precision (6–7 nm; Fig. 1a,b), the average Brp cluster size indeed very closely matches the diameter of CAZ-filaments (see Discussion section).

### Quantifying the substructural organization of Brp in the CAZ

**Figure 3:**
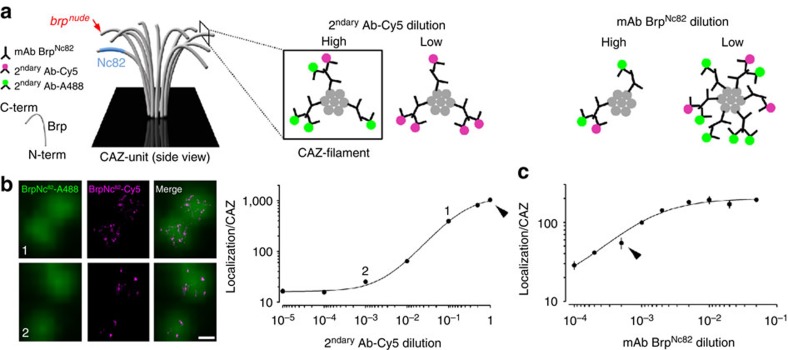
Quantification of Brp molecules in the CAZ. (**a**) Schematic description of the experiments. A filamentous CAZ-unit is shown in grey and the polarized orientation of Brp is illustrated. The blue shaded area indicates the approximate region of the mAb Brp^Nc82^ epitope, the red arrow marks the C-terminal (C-term) truncation in *brp*^*nude*^. (**b**) Cy5 and A488 labelled 2^ndary^ F(ab′)_2_ fragments were diluted at a constant overall concentration of 5.2 × 10^−8^ M (for example, 1: 100% Cy5 to 0% A488 and 10^−3^: 0.1% Cy5 to 99.9% A488) and a fixed mAb Brp^Nc82^ dilution (1/2,000; 0.5 × 10^−3^) to estimate the number of localizations corresponding to a single Cy5-labelled antibody attached to Brp via mAb Brp^Nc82^. Individual Brp proteins are indicated by filled circles (grey). Binding of single Cy5 antibodies (magenta) to the CAZ was verified through comparison with the A488 epifluorescence signal (green; see Methods section). Images depict examples of several antibody dilutions (indicated by 1 and 2 in the graph). (**c**) Titration of mAb Brp^Nc82^ at fixed Cy5- and A488 antibody concentration (5.2 × 10^−8^ M; 1% Cy5, 99% A488) provides information on epitope saturation by the primary antibody. Data in (**b**) (*n*=8–10 NMJs per dilution) and (**c**) (*n*=4–5 NMJs per dilution, error bars show s.e.m.) were fit to a logistic function (see Methods section). Arrowheads denote antibody concentrations used in experiments (Figs 1, 2, 4 and ). Scale bar, 200 nm. N-term, N terminus.

To attain further quantitative information on the molecular AZ architecture, we developed an approach to count Brp molecules in their native environment. Owing to the single-molecule sensitivity of *d*STORM, fluorophore localizations can be used not only to extract information about the distribution of individual molecules^30^, but crucially, also to approximate the absolute number of protein copies. Since the number of localizations measured for a fluorophore-labelled antibody is influenced by its nano-environment, reference experiments using different antibody concentrations had to be conducted at the CAZ to unequivocally correlate localization counts with the number of underlying Brp protein epitopes (Fig. 3; see Methods section).

First, titrations were performed with secondary antibodies to unravel the number of localizations detected for a single Cy5-labelled secondary antibody (2^ndary^ Ab-Cy5) bound to the primary antibody within the CAZ (Fig. 3b). In order to reliably identify the CAZ at low 2^ndary^ Ab-Cy5 concentrations (high dilutions), mAb Brp^Nc82^ was co-stained with Alexa Fluor 488 (2^ndary^ Ab-A488). Next, the concentration of the primary antibody was titrated to estimate saturation of Brp epitopes (Fig. 3c). And finally, the number of secondary antibodies bound to each primary antibody was estimated. To obtain this information, a low concentration of mAb Brp^Nc82^ was combined with a normal concentration of 2^ndary^ Ab-Cy5. Comparing the number of Cy5 localizations per putative Brp epitope with those of a single 2^ndary^ Ab-Cy5 (Fig. 3b) provided an approximation of 1.59 2^ndary^ Ab-Cy5 per mAb Brp^Nc82^. To visualize the CAZ in this experiment, co-staining was performed with an antibody directed against an N-terminal Brp epitope (rabbit-Brp^N-term^ plus 2^ndary^ Ab-A488 anti-rabbit)^20^.

Taking these considerations into account allows for quantitative image analysis and delivers an estimate of 137±29 (s.e.m.) Brp molecules per CAZ-unit (conversion factor of molecules per localization: 0.134±0.028 s.e.m.; see Methods section). Correspondingly, ~7 Brp molecules are recognized per multiprotein cluster (52.2±0.7 localizations s.e.m., *n*=2,102 clusters). According to this calculation, Brp proteins would assemble as polarized rod-like heptamers via coiled-coils to form a multiprotein filament. The plausibility of this stoichiometry can be appreciated by comparison with other filamentous protein structures, for example, seven subfibrils form intermediate filaments of ~10 nm diameter^31^.

Interestingly, the image analysis described that ~26% of Brp localizations in CAZ-units are not clearly grouped into clusters (Fig. 2d, black triangles). Taking the image background into account (only 78±7 localizations per μm^2^ s.e.m.), we estimate that <1% of localizations within CAZ-units (~10^4^ localizations per μm^2^) are caused by unspecific labelling. This indicates that a substantial fraction of Brp molecules are not part of macromolecular filaments.

In localization microscopy, labelling density, in addition to localization precision, critically determines the ability to resolve spatial features of a given structure^32^. Thus, the high density of Brp molecules at the CAZ and the strong affinity of the antibodies form the basis for the high spatial resolution in *d*STORM experiments. While epitope shielding may well introduce an error to our approximation of Brp protein and cluster numbers, the tight correlation with EM data lends strong support to our quantitative approach.

### Ultrastructural analysis of different AZ states

Brp reorganizations are involved in synaptic plasticity operating on time scales ranging from milliseconds to days^5^,^6^,^17^,^33^. Such plastic rearrangements could, in principle, involve changes in Brp protein number per CAZ^34^ or the spatial orientation of Brp within the CAZ^17^. To test our quantitative imaging approach, we analysed different AZ states by using two previously investigated *Drosophila* mutants with altered Brp organization.

The small vesicle-associated GTPase Rab3 regulates the enrichment of Brp at AZs. At Rab3 mutant (*rab3*^*rup*^) larval NMJs, altered synaptic transmission is accompanied by fewer Brp-positive synapses, although these display greatly enlarged Brp aggregates^34^. The hypomorphic mutant, *brp*^*nude*^, lacks merely the last 17 C-terminal amino acids of Brp (that is, ~1% of the entire protein; Fig. 3a). While the overall CAZ structure is left intact, *brp*^*nude*^ T-bars display a strikingly reduced vesicle tethering capacity that leads to slowed vesicle recruitment and short-term depression of neurotransmitter release^17^.

**Figure 4:**
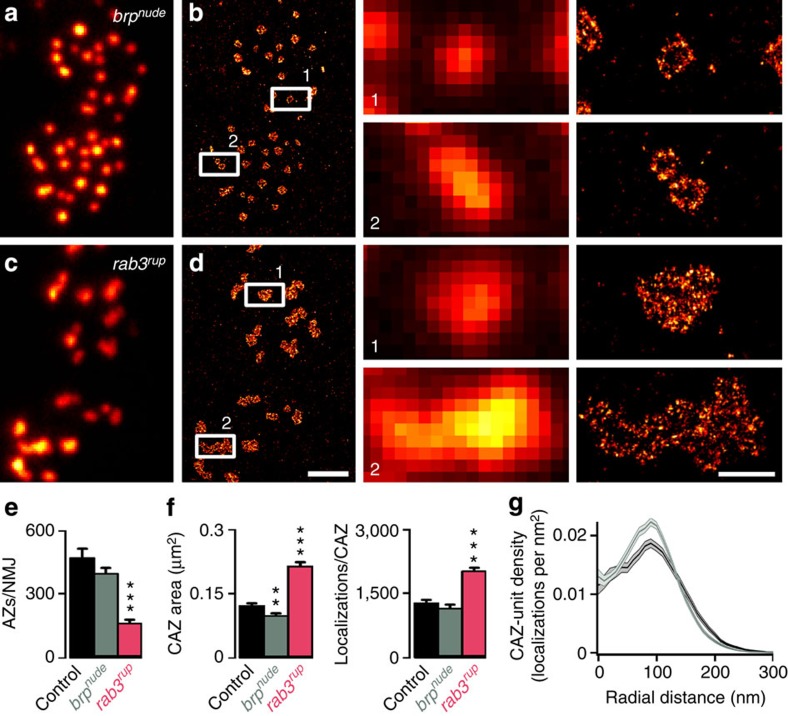
Variable nano-organization of Brp in the CAZ. (**a**,**b**) Overview of *brp*^*nude*^ and (**c**,**d**) *rab3*^*rup*^ NMJs stained against Brp. Enlarged boxed regions (left panels epifluorescence and right panels *d*STORM) demonstrate the ordered arrangement of *brp*^*nude*^ CAZs, with Brp immunoreactivity largely confined to the CAZ-unit margins, and the disordered nanoscopic organization of greatly enlarged *rab3*^*rup*^ CAZs. (**e**–**g**) Quantification of imaging data acquired with confocal (**e**, rank sum test versus controls (*n*=14 NMJs); *brp*^*nude*^ (*n*=13) *P*=0.254; *rab3*^*rup*^ (*n*=14) *P*<0.001) and localization microscopy (**f**, rank sum test versus controls (*n*=16 NMJs): *brp*^*nude*^ (*n*=13) *P*=0.005, *rab3*^*rup*^ (*n*=11) *P*<0.001; **g**, rank sum test versus controls (*n*=16 NMJs): *brp*^*nude*^ (*n*=13) *P*=0.28, *rab3*^*rup*^ (*n*=11) *P*<0.001). (**g**) *En face* views of individual CAZ-units were aligned according to their centres of mass and the radial density distributions of Brp localizations were plotted (dark lines: average and shaded area: s.e.m.). Compared with controls (black), the Brp epitope was distributed more narrowly in *brp*^*nude*^ (grey) CAZ-units. Scale bars, 2 μm (**a**–**d**) and 500 nm (enlarged boxed regions).

In line with previous work, we used confocal microscopy to confirm that *rab3*^*rup*^ NMJs contain fewer Brp-positive AZs (~35% of controls; control: 472±43 s.e.m., *n*=14 NMJs; *rab3*^*rup*^: 164±15, *n*=14, rank sum test *P*<0.001; Fig. 4e and Table 1; ref. 34) and then applied *d*STORM to study the nanoscopic organization of Brp at individual AZs. In *rab3*^*rup*^ mutants, the CAZ was significantly enlarged (control: 0.120±0.006 μm^2^ s.e.m., *n*=16 NMJs; *rab3*^*rup*^: 0.212±0.01 μm^2^, *n*=11, rank sum test *P*<0.001; Fig. 4f and Table 1) and contained more Brp molecules (control: 1,257±89 localizations s.e.m., *n*=16 NMJs; *rab3*^*rup*^: 1,999±98, *n*=11, rank sum test *P*<0.001; Fig. 4f and Table 1). Electron micrographs have described a high concentration of T-bars at a subpopulation of *rab3*^*rup*^ AZs, which likely correspond to those AZs that are Brp-positive^34^. In addition, *d*STORM resolved a complex organization of the large *rab3*^*rup*^ CAZ, often lacking a clearly distinguishable modular composition (Fig. 4, enlarged boxed regions, lower panel). CAZ-units could therefore not be unequivocally identified at *rab3*^*rup*^ AZs.

In contrast, *brp*^*nude*^ NMJs contained normal numbers of Brp-positive AZs (*brp*^*nude*^: 397±25, *n*=13, rank sum test *P*=0.254 versus control; Fig. 4e and Table 1; ref. 17) and displayed a modular arrangement of Brp into units within the CAZ (Fig. 4, enlarged boxed regions upper panel). Interestingly, *d*STORM revealed a decrease in the area of the CAZ at *brp*^*nude*^ AZs (0.097±0.005 μm^2^ s.e.m., *n*=13 NMJs, rank sum test *P*=0.005 versus control; Fig. 4f and Table 1). This structural property had heretofore not been recognized using high-resolution imaging via STED or EM^17^. Despite this decrease in size, the average *brp*^*nude*^ CAZ contained normal numbers of Brp molecules (1,129±104 localizations s.e.m., *n*=13 NMJs, rank sum test *P*=0.28 versus control; Fig. 4f and Table 1). This prompted us to investigate the spatial organization of Brp in more detail. Because the arrangement of Brp within individual *brp*^*nude*^ CAZ-units appeared very ordered, we analysed its radial distribution. We found that the Brp epitope is more confined to the perimeter at *brp*^*nude*^ CAZ-units and more uniformly distributed in controls (Fig. 4g).

### Functional properties of AZ states

**Figure 5:**
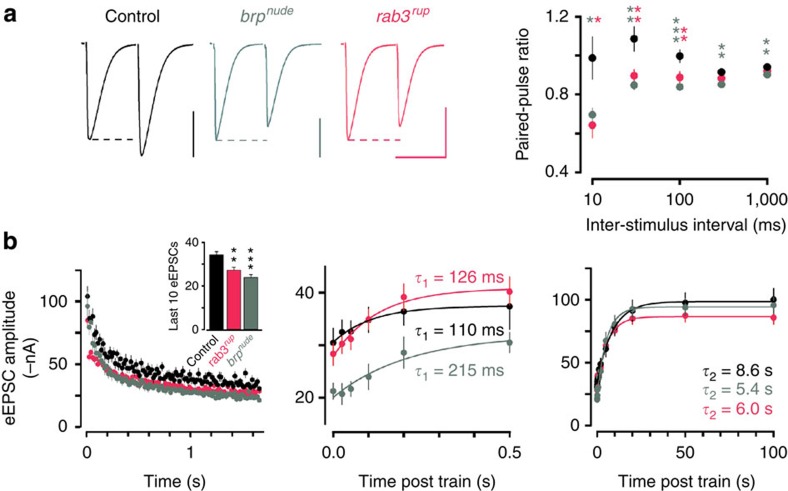
Electrophysiological characterization of different AZ states. (**a**) Representative traces (normalized amplitude of the first eEPSC) and average data of two-electrode voltage clamp recordings from larval NMJs show similar depression of *brp*^*nude*^ (grey) and *rab3*^*rup*^ (pink) eEPSCs during paired-pulse stimulation (rank sum test versus controls: *brp*^*nude*^ for 10 ms *P*=0.014, for all other intervals *P*≤0.01; *rab3*^*rup*^ for 10 ms *P*=0.016, for 30 and 100 ms *P*≤0.01) and (**b**) 60 Hz trains (eEPSC 91–100 mean; control: −34.3±1.5 nA s.e.m., *n*=10 NMJs; *brp*^*nude*^: −23.9±1.3 nA, *n*=10, rank sum test *P*<0.001 versus control; *rab3*^*rup*^: −27.3±1.4 nA, *n*=11, *P*=0.004 versus control). The fast phase of recovery after stimulation is selectively slowed in *brp*^*nude*^ (centre panel), while all three genotypes show a normal slow recovery phase (right). Scale bars, (**a**) 30 nA, 30 ms. Data are presented as mean±s.e.m.

Next, we performed electrophysiological recordings to obtain mechanistic descriptions of AZ function in both mutants. During low levels of activity, synaptic transmission is fairly normal at both *rab3*^*rup*^ and *brp*^*nude*^ NMJs (Supplementary Figs 2 and 3)^17^,^34^. While both paired-pulse stimulation (Fig. 5a) and high-frequency trains of activity (Fig. 5b) provoked similarly pronounced short-term depression of evoked excitatory postsynaptic currents (eEPSCs) in both mutants, closer inspection revealed subtle differences in short-term plasticity (STP). Whereas *rab3*^*rup*^ NMJs showed normal biphasic recovery kinetics, *brp*^*nude*^ synapses displayed a slow initial phase of recovery, characteristic of slow vesicle recruitment (Fig. 5b)^17^. These dissimilar properties of recovery indicate different origins of depression at *brp*^*nude*^ and *rab3*^*rup*^ synapses.

**Figure 6:**
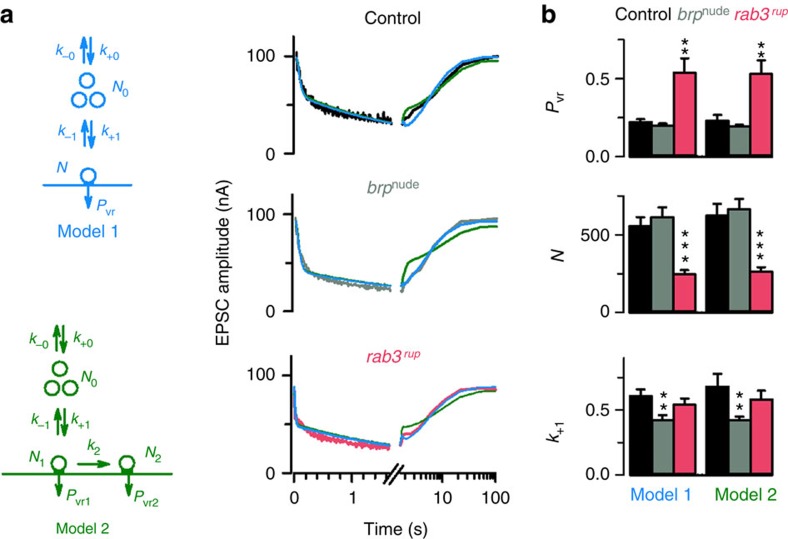
Mechanistic interpretation of AZ function. (**a**) Model 1 (blue; encompassing one pool of RRVs refilled from a finite supply pool) and model 2 (green; containing two pools of RRVs with different release probabilities) were used to describe the experimental data (example fits to average data in right panel). (**b**) The release parameters, obtained by fitting individual trains plus recovery experiments (control: *n*=10, *brp*^*nude*^: *n*=10 and *rab3*^*rup*^: *n*=11), distinguish between *brp*^*nude*^ and *rab3*^*rup*^ phenotypes. Statistics used Kruskal–Wallis with Dunn’s multiple comparison tests. Plots show mean±s.e.m.

To describe synaptic depression in quantitative terms of the number of RRVs (*N*), their release probability (*p*_vr_) and the rate of vesicle reloading at release sites (*k*_*+*1_), an established modelling approach was used (Fig. 6; see Methods section)^5^,^18^. Two constrained STP models were used to reproduce individual high-frequency trains with ensuing biphasic recovery. For *brp*^*nude*^ NMJs, both models ascribed short-term depression to (i) a decreased rate of replenishing (ii) a regular number of RRVs (iii) possessing a normal average release probability (Fig. 6b and Supplementary Table 1). This is related to (i) impaired vesicle tethering to the CAZ, and agrees with (ii) a normal electrophysiologically measured RRV pool size and (iii) regular calcium channel clusters at *brp*^*nude*^ AZs^17^.

Consistent with previous interpretations^34^,^35^, both models predicted few RRVs possessing a high *p*_*vr*_ at *rab3*^*rup*^ NMJs (Fig. 6b and Supplementary Table 1). Despite normal vesicle reloading rates, this setting is susceptible to short-term depression owing to the depletion of a large RRV fraction at stimulus onset.

In principle, short-term depression may be provoked by a range of pre- and postsynaptic factors^36^,^37^,^38^. However, the established information on these two specific mutants^17^,^34^,^35^ and a previous analysis of postsynaptic properties^18^ allowed us to focus on the recruitment of RRVs to the AZ and the availability of release sites as rate-limiting factors of synaptic transmission.

### Dissecting structure–function relationships

Building upon the mutant analyses of different AZ states, we sought to link quantitative information on the CAZ ultrastructure with functional properties of neurotransmitter release. In view of the modelling results, the number of Brp-positive AZs per NMJ (~35% of controls in *rab3*^*rup*^; Fig. 4e) scales roughly with the number of RRVs (Fig. 6b). Such a calculation predicts on average ~3 RRVs per AZ in all three genotypes (wt ~2.5, *brp*^*nude*^ ~3.2, *rab3*^*rup*^ ~3.1; see Methods section). Hence, in terms of RRVs, the almost twofold larger *rab3*^*rup*^ CAZ cannot compensate for reduced AZ numbers.

Since Brp contributes to clustering Ca^2+^ channels at the AZ^16^, the increased recruitment of Brp proteins to the *rab3*^*rup*^ CAZ (Fig. 4f) will likely also increase the recruitment of Ca^2+^ channels and may tighten the spatial coupling to RRVs. This will elevate *p*_vr_, consistent with the modelling results (Fig. 6b) and the larger Ca^2+^ channel clusters reported at *rab3*^*rup*^ AZs^34^.

**Figure 7:**
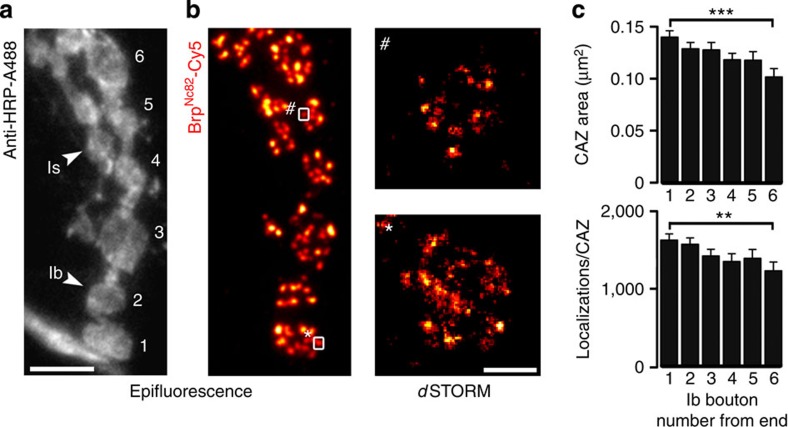
Neuronal gradient of the CAZ ultrastructure. (**a**,**b**) Epifluorescence dual-channel images.(**a**) Staining against horseradish peroxidase (anti-HRP, grey) displays type Is and type Ib motorneurons (indicated by arrowheads) at a wt NMJ. Ib boutons are numbered beginning at the distal end (in the lower left corner a neurite passes by the NMJ). (**b**) Corresponding Brp signal, which could be clearly allotted to a specific bouton. Enlarged boxed regions show examples of CAZs imaged with *d*STORM. (**c**) Quantification of the CAZ ultrastructure uncovers gradients in CAZ size (Ib distal: 0.136±0.006 μm^2^ s.e.m., *n*=269 CAZs; Ib proximal: 0.099±0.008 μm^2^ s.e.m., *n*=105, rank sum test *P*<0.001) and Brp localizations per CAZ (Ib distal: 1,583±77 localizations s.e.m., *n*=269 CAZs; Ib proximal: 1,200±110 localizations s.e.m., *n*=105, rank sum test *P*=0.003) along type Ib motorneurons, which closely match the functional gradient^35^,^39^. Scale bars, 5 μm (**a**,**b**) and 200 nm (enlarged boxed regions). Data are presented as mean±s.e.m.

To further test this structure–function interpretation, which was reached by studying mutant AZ states, we turned to an intrinsic physiological property of the *Drosophila* NMJ. Two glutamatergic motorneurons, containing either small (type Is) or big (type Ib) boutons, innervate larval muscles^28^. Previous studies have described a functional gradient along the type Ib motorneuron, with larger Ca^2+^ signals and correspondingly higher *p*_vr_ at the terminal bouton despite a constant density of AZs^35^,^39^. A structural correlate of this gradient has, however, not been identified. Hence, we used *d*STORM to study the ultrastructural organization of Brp at wt motorneurons. Our data resolve a clear gradient of both CAZ size and the number of Brp molecules per CAZ along the Ib neuron (Fig. 7). With the largest values at terminal boutons, this structural diversity closely matches the functional gradient^35^,^39^.

## Discussion

Here, we introduce a novel procedure to determine endogenous protein numbers in tissue by localization microscopy using standard labelling with primary antibodies and secondary F(ab′)_2_ fragments. Correlating the nanoscopic organization of a single large protein with electrophysiological recordings enabled us to link the filamentous CAZ ultrastructure to neurotransmitter release properties.

Our data are in line with the observation that *p*_vr_ scales with AZ size^40^. Studies at *Drosophila* NMJ and hippocampal synapses have also reported that larger AZs provide for more RRVs^5^,^40^,^41^. Surprisingly, our analysis of the grossly enlarged *rab3*^*rup*^ CAZ does not support this notion. A possible explanation could be provided by the disordered accumulation of Brp at the *rab3*^*rup*^ CAZ (Fig. 4, enlarged boxed regions, lower panel) that suffices to increase *p*_vr_ via calcium channel clustering but fails to provide additional vesicle release sites. However, keeping in mind that release persists and *N* appears unaltered in *brp*-null mutants, Brp itself is unlikely the primary determinant of a release site^16^,^18^. In any case, it will be of great interest to clarify why the wt CAZ is subdivided into Brp-positive CAZ-units, and to investigate whether this modular arrangement is matched by a complementary substructural organization of the opposing postsynaptic density.

Our results demonstrate that counting Brp proteins and quantifying their spatial distribution provides more detailed and precise information than merely measuring the CAZ area. This is exemplified by the small *brp*^*nude*^ CAZ that contains a normal number of Brp localizations and gives rise to an unchanged *p*_vr_ (Figs 4f and 6b and Table 1). Instead, Brp localizations are more confined to the perimeter of *brp*^*nude*^ CAZ-units (Fig. 4g). Altered post-translational modification of Brp can promote vesicle tethering to the CAZ and provokes spreading out of AZ-filaments^42^. Conversely, the small *brp*^*nude*^ CAZ correlates with deficient vesicle tethering and slow vesicle recruitment, leading to short-term depression (Figs 5 and 6)^17^. Hence, vesicle tethering may contribute to the shape of the CAZ by contorting proteinaceous filaments or, alternatively, the precise CAZ conformation affects its tethering ability. While this cannot be specified at present, either way the spatial organization of Brp provides information on vesicle reloading kinetics.

By engaging *d*STORM, we discovered a heretofore unrecognized gradient of the CAZ ultrastructure along a glutamatergic neuron, concealed by the limited resolution of confocal microscopy^35^. Importantly, this finding provides a mechanistic basis for the functional diversification of AZs^35^,^39^. A functional gradient has not been described for type Is motorneurons. Moreover, AZs of Is neurons reportedly possess a higher *p*_vr_ than their Ib counterparts, although this functional feature is not matched by a larger number of T-bars per AZ^28^,^29^,^43^. Similarly, we detected comparable numbers of Brp localizations at type Is and Ib CAZs (the Is CAZ is in fact slightly smaller) and found no ultrastructural gradient in the Is neuron (Supplementary Fig. 4). This consideration highlights that Brp is not the sole determinant of *p*_vr_ and motivates the study of further AZ protein constituents. Intriguingly, for example, synaptic vesicle size differs between these two different glutamatergic neurons^44^.

The present investigation emphasizes how fundamentally different AZ states may be disguised by giving rise to similar facets of short-term depression (Fig. 5a). For descriptions of synaptic function, the degree of paired-pulse depression is routinely interpreted to reflect the magnitude of release probability. In light of these results and recent data from different synapses reporting fast vesicle reloading^45^, transient fusion^46^ and release site clearance^47^, analysing the CAZ nanostructure to test alternative interpretations of depression may well provide new insights into the molecular control of neurotransmission.

In order to estimate the number of Brp molecules in the CAZ, several hurdles had to be overcome. In general, localization microscopy in combination with photoactivatable fluorescent proteins^10^,^12^ appears to represent the method of choice for quantification purposes^48^ because fluorescent proteins offer the distinct advantage of specific stoichiometric labelling of target molecules. On the other hand, we were interested in endogenous protein levels. Therefore, overexpression of fusion proteins could not be deployed, and substitution of native proteins by transgenic variants that display wt expression and function remains challenging. In addition, misfolded fluorescent proteins and those that cannot be photoactivated or photobleached already after only a few excitation/emission cycles, and thus emit an insufficient number of photons, withdraw themselves from detection and localization^14^,^49^.

Besides localization microscopy, stepwise stochastic photobleaching of fluorophores upon illumination with light can be used to determine protein numbers^50^. Stepwise photobleaching is, however, limited to low protein numbers because the likelihood of missed events increases exponentially with the number of molecules^51^.

As an alternative approach, we evaluated the use of standard immunocytochemistry, that is, organic fluorophores and antibodies for quantification of endogenous protein levels. Here, challenges to be accepted include epitope accessibility, antibody affinity and multiple localizations owing to on/off switching on expanded time scales. Brp proteins appear to oligomerize as coiled-coils to form elongated, polarized filaments^20^. Therefore, it is conceivable that such a structural organization leads to a separation of epitopes along the filament circumference, promoting antibody accessibility despite a high density of Brp proteins (Supplementary Fig. 5). That said, differences in steric hindrance may exist at the level of individual AZs and in different genotypes. Furthermore, since epitopes can be shielded or lost during fixation, the determined number of Brp proteins might well represent a lower estimate.

Organic fluorophores exhibit certain advantageous characteristics, such as higher brightness and photostability than fluorescent proteins. Thus, in combination with the fact that each fluorophore can be localized multiple times, a higher percentage of accurately localized fluorophores can potentially be achieved. Nonetheless, the difficulty of extracting reliable information on how often a fluorophore-labelled antibody is localized remains, especially because the photoswitching performance of fluorophores is sensitively influenced by their local environment^52^. As a consequence, isolated fluorophores located outside of the investigated cellular compartment cannot be used as reference. Therefore, we developed an elaborate but secure two-colour method for identifying individual fluorophore-labelled antibodies in the structure of interest in order to determine the typical number of localizations. However, certain photophysical effects that depend, for example, on local fluorophore densities and photoswitching characteristics can never be completely ruled out in quantitative localization microscopy experiments. Nevertheless, by titrating primary and secondary antibodies, the described procedure delivers reliable estimates for the number of accessible protein epitopes per CAZ.

A major challenge facing the field of super-resolution microscopy is the development of analytical tools to quantify data sets and to help provide biological interpretations^30^. The implementation of clustering algorithms provided an objective description of the distribution pattern of Brp molecules within the CAZ and revealed the organization of Brp into supramolecular clusters. Considering their structural properties, these clusters likely correspond to the multiprotein CAZ-filaments observed in EM^27^ (STED microscopy displays ~9 ‘dots’ per AZ^41^). Why are the clusters elliptical? We speculate that an answer can be provided by the arrangement of fluorophores around CAZ-filaments in space. When CAZ-units are viewed *en face* (optical axis perpendicular to AZ membrane), filaments bent outwards could be viewed at a right angle to their long axis at the level of the mAb Brp^Nc82^ epitope (Fig. 3a and Supplementary Fig. 5). In the images, the filament diameter (~10 nm) only contributes to the separation of encircling fluorophores in x and y. Hence, the largest separation will be seen for Cy5 molecules located at opposite sides of the filament and aligned with the CAZ-unit circumference (Supplementary Fig. 5).

Intriguingly, the quantitative analysis described a substantial population of un-clustered Brp proteins in the CAZ. It will be of great interest to investigate the biological significance of this observation and to test whether, for example, the fraction of ‘free’ Brp proteins changes during synaptic activity.

The complex nanoscopic organization and highly dynamic interactions of AZ proteins pose a formidable challenge to deciphering structure–function relationships of neurotransmission. A synergy of sophisticated biochemistry^53^,^54^ with super-resolution microscopy^55^ holds great promise for assembling a comprehensive molecular blueprint of the AZ.

## Methods

### Fly stocks

The following genotypes were used in this study: *w*^*1*^ or *w*^*1118*^ (controls), *brp*^*nude(5.38)*^/*df(2R)BSC29* (*brp*^*nude*^)^17^ and *rab3*^*rup*^/*df(2R)ED2076* (*rab3*^*rup*^)^34^. Data were obtained from male third instar *Drosophila* larvae raised at 25 °C.

### Electrophysiology

In brief, two-electrode voltage clamp recordings (Axoclamp 900A amplifier, Molecular Devices) of eEPSCs (*V*_holding_ −60 mV, stimulation artefact removed for clarity in figures) and minis (*V*_holding_ −80 mV, 90 s recording) were made from muscle 6 (segments A2 and A3) at room temperature with intracellular electrodes (resistances of 10–20 MΩ, filled with 3 M KCl) essentially as previously reported^18^. The composition of the extracellular haemolymph-like solution (HL-3)^56^ was (in mM): NaCl 70, KCl 5, MgCl_2_ 20, NaHCO_3_ 10, trehalose 5, sucrose 115, HEPES 5 and CaCl_2_ 1.5, pH adjusted to 7.2. Muscle cells with an initial membrane potential between −50 and −70 mV, and input resistances of ≥4 MΩ were accepted for analysis. Signals were sampled at 10 kHz, low-pass filtered at 1 kHz and analysed with Clampfit 10.2 (Molecular Devices). EPSCs were evoked by stimulating the innervating nerve (300 μs pulses typically at 10 V) via a suction electrode.

Ten eEPSCs were averaged per cell for each paired-pulse interval and for low-frequency stimulation. Paired-pulse recordings were made at 0.2 Hz with inter-stimulus intervals of (in ms): 10, 30, 100, 300 and 1,000. Ten seconds of rest were afforded to the cell in between recordings. The amplitude of the second response in 10 ms inter-pulse recordings was measured from the peak to the point of interception with the extrapolated first response. High-frequency stimulation followed an established protocol^18^,^57^ consisting of 100 pulses applied at 60 Hz. The recovery was monitored by stimulating at increasing intervals following the train (in ms): 25, 50, 100, 200, 500, 1,000, 2,000, 5,000, 10,000, 20,000, 50,000 and 100,000.

### Modelling

STP modelling of 60 Hz trains and of the recovery thereafter was performed as previously described^5^,^18^. Two constrained STP models were used. First, a model with one pool of release-ready vesicles refilled from a finite supply pool was used (model 1). Model 1 is characterized by the following parameters: γ, the Ca^2+^ dependence of facilitation; *α*, that defines the release probability; *N*, the number of RRVs; *N*_*0*_, the number of vesicles in the supply pool from which the readily releasable pool is refilled; and *k*_*+*1_, *k*_-1_, *k*_*+*0_ and *k*_-0_, the refilling rates of *N* and *N*_0_, respectively. In addition, we also used a model with two pools of release-ready vesicles and heterogeneous release probabilities (model 2). In model 2, a small pool of release-ready vesicles (*N*_2_) with a high release probability (*p*_vr2_) is refilled with a rate *k*_2_ from a larger pool (*N*_1_), which has a lower vesicular release probability (*p*_vr1_), and which, in turn, is refilled from a supply pool (*N*_0_). The refilling rates of these pools (*k*_+1_, *k*_−1_, *k*_+0_ and *k*_−0_) are defined as in model 1.

The release probabilities (*p*_vr_ for model 1, and *p*_vr1_ and *p*_vr2_ for model 2) were defined according to a biophysical Ca^2+^-dependent model of facilitation with one single free parameter (*α*)^18^,^58^. Both models had two additional free parameters: *N* for model 1, *N*_1_ for model 2 and *k*_+1_ for both models, resulting in three free parameters for each model. The remaining parameters were constrained to values previously estimated at the *Drosophila* NMJ^18^ with the facilitation parameter (*γ*)^58^ adjusted to 0.4 μm^−1^ to reproduce the initial facilitation observed here with an extracellular Ca^2+^ concentration of 1.5 mM.

Individual experiments including depression during 60 Hz train stimulation and the recovery from depression were fitted with either models 1 or 2 as previously described^18^. The best-fit parameters for both models are shown for each individual experiment of the different genotypes as mean and s.e.m. in Fig. 6b.

### Confocal imaging

Larvae were dissected in ice-cold HL-3, fixed for 10 min using 4% paraformaldehyde in 0.1 M phosphate-buffered saline (PBS) and blocked for 30 min in PBT (PBS with 0.05% Triton X-100, Sigma) containing 5% normal goat serum (Jackson ImmunoResearch). Preparations were incubated with primary antibodies at 4 °C overnight. After one short and three × 20 min washing steps, the filets were incubated with secondary antibodies for 2 h followed by another three washing steps. The samples were mounted in Vectashield (Vector Laboratories) for confocal imaging or kept in PBT for *d*STORM measurements. Primary antibodies were used in the following dilutions: monoclonal antibody (mAb) Brp^Nc82^ (1:250, provided by E. Buchner) and rabbit-GluRIID (1:1,000, provided by S.J. Sigrist). Alexa Fluor 488-conjugated mouse (Invitrogen) and Cy3-conjugated rabbit (Dianova) antibodies were used at 1:250. Images were acquired with a Zeiss LSM5 Pascal confocal system (objective: 63 × , numerical aperture 1.25, oil). For each set of experiments, all genotypes were stained in the same vial and imaged in one session. To estimate synapse numbers laser power was adjusted individually for each NMJ.

Brp punctae and GluRIID clusters (NMJ 6/7, segments A2, A3) were examined using ImageJ software (National Institutes of Health) in principle as previously described^6^. After background subtraction, a Gaussian blur (0.9 px s.d.) was applied to maximum z-projections of confocal stacks and masks were generated (threshold mean grey value of 25 for Brp and 30 for GluRIID). After superimposing the binary mask on the original blurred image, spot detection and segmentation via the ‘Find Maxima’ operation was performed to extract particle numbers.

To estimate the number of release sites (*N*) per AZ, the modelling prediction of *N* (average value of both models) was divided by the number of AZs on muscle 6 identified in confocal images, that is, half of NMJ 6/7 (ref. 28).

### Super-resolution imaging

The mAb Brp^Nc82^ was used at a dilution of 1/2,000 to identify AZs. Goat anti mouse F(ab′)_2_ fragments (A10534, Invitrogen) were labelled with Cy5-NHS (PA15101, GE Healthcare) according to standard coupling protocols given by the supplier. Purification of the conjugates was performed by use of gel filtration columns (Sephadex G-25, GE Healthcare). The degree of labelling was determined by absorption spectroscopy (Jasco) as 1.3 for studies of the CAZ ultrastructure and 1.3–1.5 for dilution experiments. Samples were stored in 0.2% sodium azide in PBS and for the experiments, Cy5-labelled secondary antibody was used at a concentration of 5.2 × 10^−8^ M.

For *d*STORM imaging with Cy5, the sample was embedded in photoswitching buffer, that is, 100 mM mercaptoethylamine, pH 8.0, enzymatic oxygen scavenger system (5% (wt/vol) glucose, 5 U ml^−1^ glucose oxidase and 100 U ml^−1^ catalase^59^) and mounted on an inverted microscope (Olympus IX-71) equipped with an oil-immersion objective (60 × , numerical aperture 1.45, Olympus) and a nosepiece stage (IX2-NPS, Olympus)^22^. For excitation of Cy5, a 641-nm diode laser (Cube 640–100C, Coherent) was used. Telescope lenses and mirror were arranged on a translation stage to allow for switching between wide-field, low-angle/highly inclined thin illumination and total internal reflection fluorescence imaging^22^,^60^,^61^.

Fluorescence light from Cy5 was filtered by a dichroic mirror (650, Semrock) and a band- and long-pass filter (BrightLine 697/75, RazorEdge 647, Semrock), and imaged on an electron-multiplying CCD camera (EMCCD; Ixon DU897, Andor Technology). Additional lenses were used to generate a final camera pixel size of 107 nm. Fifteen thousand frames were recorded with a frame rate of 100 Hz at an irradiation intensity of ~5 kW cm^−2^. For imaging A488, a 488-nm laser (Sapphire 488 LP, Coherent) and a polychromatic dichroic mirror (410/504/582/669, Semrock) were used. Fluorescence light from A488 was reflected by a dichroic mirror (630 DCXR, Chroma) and imaged on a second EMCCD camera equipped with a band-pass filter (HQ535/50, Chroma).

Goat anti mouse IgG labelled with A532 (A11002, Invitrogen) and A700 (A21036, Invitrogen) was used at a concentration of 6.25 × 10^−9^ M. The degree of labelling was determined as 2.0 (A700) and 4.5 (A532). Imaging of A532 and A700 by *d*STORM was performed in PBS containing 100 mM mercaptoethylamine, pH 8.3. Using appropriate filter sets (dichroic mirrors: 650 or 545/650; band-pass filters: RazorEdge 647 or BrightLine 582/75, Semrock), the samples were irradiated at 641 nm (A700) or 532 nm (NANO 250-532-100, Linos; A532) at ~5 kW cm^−2^. For titrations of A532-labelled secondary antibodies (Supplementary Fig. 6), fluorescence light from A700 and A532 was separated by a dichroic mirror (630 DCXR, Chroma) and imaged on two EMCCD cameras.

Super-resolution images were reconstructed using the software package rapi*d*STORM^62^,^63^. Only fluorescence spots containing >1,000 photons were analysed. Double-spot emission was analysed by a two-kernel analysis as described^64^ applying a maximum two-kernel improvement of 0.1. Raw localization data obtained from rapi*d*STORM was examined and further processed with ImageJ. A subpixel binning of 10 nm px^−1^ was applied. Representative images in Fig. 7 and magnified views in Figs 1 and 4 are shown with 7 nm binning for clarity.

To measure CAZ (defined by Brp^Nc82^) area and localization numbers, masks were created by applying a Gaussian blur (1 px s.d.) followed by a minimal threshold (0.15 counts). After a minimum overlay of the original data with the masks, CAZs were then identified via their area (300 px to infinity). For the comparison of genotypes, a total of 812 CAZs in controls, 776 in *brp*^*nude*^ and 257 in *rab3*^*rup*^ were analysed and data (presented as mean±s.e.m.) were acquired in two imaging sessions, each containing all three genotypes stained in the same vial. Images with a background of >2.3 single spots per μm^2^ were excluded from the comparative analysis. Unspecific background labels exhibited equal localization counts in all genotypes (average counts control: 12.0±0.2 localizations s.e.m., *n*=16 NMJs; *brp*^*nude*^: 12.0±0.1, *n*=13; *rab3*^*rup*^: 12.4±0.3, *n*=11), indicating comparable imaging settings.

For the investigation of different motorneurons, a total of 963 (type Ib) and 579 (type Is) CAZs (from NMJ 6/7, segments A2 and A3) were analysed to determine the gradient. Double-stainings included horseradish peroxidase directly conjugated to A488 (1:250, Jackson ImmunoResearch) for visualization of boutons. In the representative images, the epifluorescence signals were background subtracted and normalized.

To specify the localization precision of *d*STORM images, localizations of unspecific background label (*n*=21,436) from all three genotypes were analysed using ImageJ. Masks were created as described above (Gaussian blur with 1 px s.d., threshold 0.08 counts). Within a *d*STORM image, only selections with a size between 16 and 100 px (10 nm px^−1^) and an ellipticity ≥0.95 were anaylzed. The coordinates of localizations within a single selection were aligned to their centre of mass and a 2D histogram of all localizations (209,537 in total) was generated (binning: 4 nm × 4 nm). A 2D Gaussian function was fitted to this histogram (adjusted *R*^2^=0.995). The s.d. of the Gaussian function (*σ*_*x*,*y*_=(*σ*_*x*_+*σ*_*y*_)/2)) was determined as 7.16±0.02 nm and is stated as localization precision in this work (Fig. 1a,b). This value is comparable to the localization precision obtained with an alternative method based on nearest neighbour analysis^26^ (Supplementary Fig. 1).

For the investigation of CAZ-units, those structures were chosen that were not grouped together and were viewed *en face*, that is, with the AZ membrane perpendicular to the optical axis^20^. For the manual selection of CAZ-units, the genotypes were blinded.

To calculate the radial distribution (Fig. 4g), Mathematica 9.0 (Wolfram Research) was used to automatically calculate the centre of each chosen CAZ-unit as the centre of mass (that is, the average localization of all pixels of the CAZ-unit weighted with the pixel value). Subsequently, the distance of each pixel to the centre of mass was calculated. These distances were then binned, the pixel values were added to the corresponding bins and the values were normalized by the area of each radial slice. The resulting distributions were averaged across all chosen CAZ-units, resulting in mean and s.e.m. values for the radial distributions of each genotype.

### Quantification of Brp protein numbers

To estimate the number of Brp molecules per CAZ-unit, a number of parameters had to be considered. First, the mAb Brp^Nc82^ specifically recognizes one epitope per Brp molecule. Second, it is unclear how many Cy5-labelled F(ab′)_2_ fragments can bind to the primary mAb Brp^Nc82^ and how many localizations can be expected per Cy5-labelled secondary antibody. Here, it has to be considered that the number of localizations detected per antibody can be strongly influenced by its nano-environment. Hence, the number of localizations expected for an individual labelled antibody should ideally be derived from measurements performed under identical imaging and buffer conditions in the same cellular environment, that is, within the CAZ. In order to derive quantitative values of Brp molecules from the localization data, we performed antibody titrations with (i) different dilutions of Cy5-labelled secondary antibody and a constant concentration of mAb Brp^Nc82^, and (ii) different dilutions of mAb Brp^Nc82^ and a constant concentration of Cy5-labelled secondary antibody.

To evaluate the localizations presented by a Cy5-labelled F(ab′)_2_ fragment attached to Brp via mAb Brp^Nc82^, the concentration of mAb Brp^Nc82^ was kept constant (1/2,000, that is, experimental concentration) and the secondary antibody was diluted (1, 1/2, 1/10, 1/100, 1/1,000, 1/10,000 and 1/100,000). Preparations were simultaneously stained with A488 goat anti mouse F(ab′)_2_ fragments (A11017, Invitrogen) to warrant an overall constant secondary antibody concentration of 5.2 × 10^−8^ M for epitope saturation (for example, 1: 100% Cy5 to 0% A488 and 1/1,000: 0.1% Cy5 to 99.9% A488) and to enable the unequivocal identification of the CAZ at low Cy5 antibody concentrations (Fig. 3b). The epifluorescence signal (A488) was background subtracted, blurred and contrast enhanced to identify Cy5 localizations within in the CAZ. NMJs (8–10) were evaluated for each dilution and the localizations per CAZ were histogrammed and fit to a Poisson model in order to extract the average number of localizations per CAZ (*L*_CAZ_) as the mean of the distribution. *L*_CAZ_ as a function of the Cy5 antibody dilution (*d*) was then approximated with the logistic function *L*_CAZ_=*L*_2_+(*L*_1_−*L*_2_)(1+(*d*/*d*_0_)^*p*^)^−1^, where the lowest localization value (*L*_2_) is equivalent to the number of localizations corresponding to a single F(ab′)_2_ fragment attached to Brp via mAb Brp^Nc82^ (*L*_1_ is the maximum localization value, *p* is the Hill coefficient, which was fixed to 1; Fig. 3b). Such titrations are not limited to Cy5 but can, in principle, also be performed with other fluorophore-labelled antibodies (Supplementary Fig. 6).

The saturation of mAb Brp^Nc82^ was analysed by using a constant concentration of secondary antibodies (5.2 × 10^−8^ M) with a fixed ratio of Cy5 and A488 F(ab′)_2_ fragments (1% Cy5: 99% A488) and by diluting the mAb Brp^Nc82^ (1/20, 1/50, 1/100, 1/200, 1/500, 1/1,000, 1/2,000, 1/5,000 and 1/10,000). The localization data was analysed (4–5 NMJs per dilution) as described for Cy5 secondary antibody dilutions (Fig. 3c; *L*_1_=198.9 and *L*_CAZ_(1/2,000)=70.6).

In order to estimate how many Cy5-labelled secondary antibodies bind per primary mAb Brp^Nc82^ under experimental conditions (5.2 × 10^−8^ M Cy5, 100%), NMJs (*n*=5) were stained with a very low concentration of mAb Brp^Nc82^ (1/20,000) together with an antibody directed against an N-terminal Brp epitope (rabbit-Brp^N-term^ 20, provided by S.J. Sigrist, 1/2,000; labelled by Alexa Fluor 488 goat anti rabbit (A11008, Invitrogen), 1/250). Only solitary spots within CAZs defined via Brp^N-term^ were measured (threshold ≥10 px), histogrammed and fit to a Poisson model to extract the number of localizations corresponding to one mAb Brp^Nc82^ (*L*_*E*_ (Nc82)).

Using the localization values (Table 2) to solve the equation


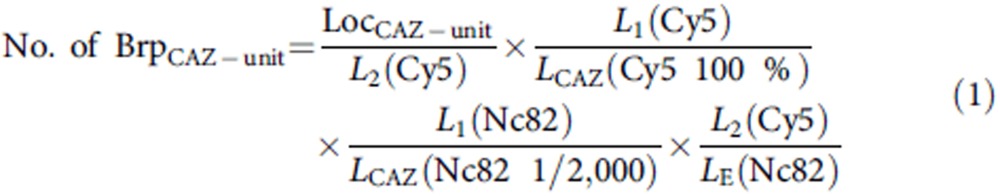


gives an estimate of 137±29 Brp molecules s.e.m. at an average CAZ-unit (we refrained from cancelling L2(Cy5) to improve the traceability of the equation). Correspondingly, the conversion factor 0.134±0.028 s.e.m. was used to translate localizations into molecules for all genotypes (Table 1).

### Cluster analysis

For the analysis, we used a home-written density-based algorithm. The base of the algorithm comes from the known algorithm, density-based spatial clustering of applications with noise: this algorithm simply looks for localizations that reside within the middle of a circle of radius Eps and enclose at least *k* other localizations^30^,^65^. Since our data contains a large number of localizations, in between putative clusters, we added more constraints on cluster detection. The algorithm starts with finding local maxima of density. The density is defined as the number of localizations within an Eps radius circle around a localization (Eps-environment). Each local maximum that has a density that is more than *k* will be defined as a cluster centre. For each cluster centre, the localizations contained within its Eps-environment are examined for holding the condition of *k* localizations. When the condition is held, the current localization along with all the other localizations within the Eps-environment will become members of this cluster. The algorithm then moves on to another localization that was found within the circle of radius Eps from the cluster centre and examines if it holds the conditions. If not, this localization will be a boundary point for the cluster and the expansion will end. If it does, this localization will be considered a core-object of the same cluster, and the cluster will keep expanding until it reaches a boundary point. In addition to the above conditions, each localization added to the cluster should have a lower density than the localization that discovered it. If the algorithm detects an increase in density, this localization will not be part of the previous cluster. This separates adjacent clusters and prevents creating saddle points between them.

We chose a search Eps of 20 nm that roughly corresponds to the estimated radius of an EM filament with the antibody complex attached to it. For a chosen Eps, the density is calculated as the number of localizations within the Eps-environment of the current localization. The parameter *k* was chosen based on the density distribution that had a peak ~12–18, and *k* was large enough to separate from noise but not too large as to find a sparse number of clusters (*k*=16).

After defining the clusters and the non-clustered localizations, we set to examine a set of different parameters that characterize the clusters. We analysed the following cluster properties: (a) the number of localizations belonging to each cluster, and (b) cluster shape and area. We found that most clusters did not show an exact circular shape but were more elliptic. Therefore, a 2D ellipse was tightly fitted to each cluster. From this ellipse, the parameters: shape (minor radius divided by major radius), minor and major axes are calculated. Clusters were defined as comprising a CAZ-unit if the density was at least four clusters within a circle of radius 200 nm.

### Statistics

Statistical tests were used as indicated. In the figures, the level of significance is denoted by asterisks: *P≤0.05, **P≤0.01, ***P≤0.001.

## Author contributions

A.D., M.H., M.S. and R.J.K. designed; and D.L., N.E., S.v.d.L. T.H. and X.Z.K. performed the experiments. A.A., A.R., M.S., N.E., S.H., R.J.K., S.v.d.L., T.H., U.A. and X.Z.K. evaluated the data. R.J.K. and M.S. supervised the project and wrote the manuscript with N.E. and contributions from A.A., A.D., A.R., M.H., S.H., S.v.d.L. and U.A.

## Additional information

**How to cite this article:** Ehmann, N. *et al*. Quantitative super-resolution imaging of Bruchpilot distinguishes active zone states. *Nat. Commun.* 5:4650 doi: 10.1038/ncomms5650 (2014).

## Supplementary Material

Supplementary Figures, Table and References Supplementary Figures 1-6, Supplementary Table 1 and Supplementary References

Supplementary Movie 1 Rotational view of a CAZ-unit. Rotation of the CAZ-unit shown in Fig. 2, displaying clusters defined by the algorithm as elevations. X and y axes are given in nm, z axis and colour code show localization density (number of localizations in 20 nm search radius, Eps environment).

## Figures and Tables

**Table 1:** Structure of AZs.

	**Confocal**	***d*****STORM**		
	**AZ/NMJ**	**Area (μm**^**2**^**)**	**Localizations**	**Number of Brp molecules**
Control CAZ	472±43 (*n*=14 NMJs)	0.120±0.006 (*n*=16 NMJs)	1,257±89	168±34
*brp*^*nude*^CAZ	397±25 (*n*=13 NMJs)	0.097±0.005 (*n*=13 NMJs)	1,129±104	151±35
*rab3*^*rup*^CAZ	164±15 (*n*=14 NMJs)	0.212±0.01 (*n*=11 NMJs)	1,999±98	268±58

AZ, active zone; Brp, Bruchpilot; CAZ, active zone cytomatrix; *d*STORM, direct stochastic optical reconstruction microscopy; NMJ, neuromuscular junction.

Confocal microscopy was used to approximate the number of AZs per NMJ (via their Brp-positive CAZ), and super-resolution imaging by *d*STORM was engaged to quantify ultrastructural properties of the CAZ and to estimate Brp protein copies. Data are presented as mean±s.e.m.

**Table 2:** Localization values from antibody titrations.

	**Loc**_**CAZ-unit**_	***L***_**2**_ **(Cy5)**	***L***_**1**_ **(Cy5)**	***L***_**CAZ**_**(Cy5 100%)**	***L***_**E**_ **(Nc82)**	***L***_**1**_ **(Nc82)**	***L***_**CAZ**_**(Nc82 1/2,000)**
Localizations	1,020.5	16.1	1,212.2	992.5	25.6	198.9	70.7
s.e.m.	42.7	1.0	117.4	100.9	1.4	6.4	7.6

CAZ, active zone cytomatrix

## References

[b1] Lichtman J. W. & Denk W. The big and the small: challenges of imaging the brain's circuits. Science 334, 618–623 (2011). 22053041 10.1126/science.1209168

[b2] Zhai R. G. & Bellen H. J. The architecture of the active zone in the presynaptic nerve terminal. Physiology (Bethesda) 19, 262–270 (2004). 15381754 10.1152/physiol.00014.2004

[b3] Südhof T. C. The presynaptic active zone. Neuron 75, 11–25 (2012). 22794257 10.1016/j.neuron.2012.06.012 PMC3743085

[b4] Atwood H. L. & Karunanithi S. Diversification of synaptic strength: presynaptic elements. Nat. Rev. Neurosci. 3, 497–516 (2002). 12094207 10.1038/nrn876

[b5] Weyhersmüller A., Hallermann S., Wagner N. & Eilers J. Rapid active zone remodeling during synaptic plasticity. J. Neurosci. 31, 6041–6052 (2011). 21508229 10.1523/JNEUROSCI.6698-10.2011 PMC6632979

[b6] Schmid A. . Activity-dependent site-specific changes of glutamate receptor composition *in vivo*. Nat. Neurosci. 11, 659–666 (2008). 18469810 10.1038/nn.2122

[b7] Matz J., Gilyan A., Kolar A., McCarvill T. & Krueger S. R. Rapid structural alterations of the active zone lead to sustained changes in neurotransmitter release. Proc. Natl Acad. Sci. USA 107, 8836–8841 (2010). 20421490 10.1073/pnas.0906087107 PMC2889309

[b8] Hell S. W. & Wichmann J. Breaking the diffraction resolution limit by stimulated emission: stimulated-emission-depletion fluorescence microscopy. Opt. Lett. 19, 780–782 (1994). 19844443 10.1364/ol.19.000780

[b9] Gustafsson M. G. Surpassing the lateral resolution limit by a factor of two using structured illumination microscopy. J. Microsc. 198, 82–87 (2000). 10810003 10.1046/j.1365-2818.2000.00710.x

[b10] Betzig E. . Imaging intracellular fluorescent proteins at nanometer resolution. Science 313, 1642–1645 (2006). 16902090 10.1126/science.1127344

[b11] Heilemann M. . Subdiffraction-resolution fluorescence imaging with conventional fluorescent probes. Angew. Chem. Int. Ed. 47, 6172–6176 (2008). 10.1002/anie.200802376 18646237

[b12] Hess S. T., Girirajan T. P. K. & Mason M. D. Ultra-high resolution imaging by fluorescence photoactivation localization microscopy. Biophys. J. 91, 4258–4272 (2006). 16980368 10.1529/biophysj.106.091116 PMC1635685

[b13] Rust M. J., Bates M. & Zhuang X. Sub-diffraction-limit imaging by stochastic optical reconstruction microscopy (STORM). Nat. Methods 3, 793–795 (2006). 16896339 10.1038/nmeth929 PMC2700296

[b14] Sauer M. Localization microscopy coming of age: from concepts to biological impact. J. Cell. Sci. 126, 3505–3513 (2013). 23950110 10.1242/jcs.123612

[b15] del Castillo J. & Katz B. Quantal components of the end-plate potential. J. Physiol. 124, 560–573 (1954). 13175199 10.1113/jphysiol.1954.sp005129 PMC1366292

[b16] Kittel R. J. . Bruchpilot promotes active zone assembly, Ca^2+^ channel clustering, and vesicle release. Science 312, 1051–1054 (2006). 16614170 10.1126/science.1126308

[b17] Hallermann S. . Naked dense bodies provoke depression. J. Neurosci. 30, 14340–14345 (2010). 20980589 10.1523/JNEUROSCI.2495-10.2010 PMC6634796

[b18] Hallermann S., Heckmann M. & Kittel R. J. Mechanisms of short-term plasticity at neuromuscular active zones of *Drosophila*. HFSP J. 4, 72–84 (2010). 20811513 10.2976/1.3338710 PMC2931299

[b19] Knapek S., Sigrist S. & Tanimoto H. Bruchpilot, a synaptic active zone protein for anesthesia-resistant memory. J. Neurosci. 31, 3453–3458 (2011). 21368057 10.1523/JNEUROSCI.2585-10.2011 PMC6623931

[b20] Fouquet W. . Maturation of active zone assembly by *Drosophila* Bruchpilot. J. Cell Biol. 186, 129–145 (2009). 19596851 10.1083/jcb.200812150 PMC2712991

[b21] Maglione M. & Sigrist S. J. Seeing the forest tree by tree: super-resolution light microscopy meets the neurosciences. Nat. Neurosci. 16, 790–797 (2013). 23799471 10.1038/nn.3403

[b22] van de Linde S. . Direct stochastic optical reconstruction microscopy with standard fluorescent probes. Nat. Protoc. 6, 991–1009 (2011). 21720313 10.1038/nprot.2011.336

[b23] Wagh D. A. . Bruchpilot, a protein with homology to ELKS/CAST, is required for structural integrity and function of synaptic active zones in *Drosophila*. Neuron 49, 833–844 (2006). 16543132 10.1016/j.neuron.2006.02.008

[b24] Weber K., Rathke P. C. & Osborn M. Cytoplasmic microtubular images in glutaraldehyde-fixed tissue culture cells by electron microscopy and by immunofluorescence microscopy. Proc. Natl Acad. Sci. USA 75, 1820–1824 (1978). 417343 10.1073/pnas.75.4.1820 PMC392432

[b25] Amiry-Moghaddam M. & Ottersen O. P. Immunogold cytochemistry in neuroscience. Nat. Neurosci. 16, 798–804 (2013). 23799472 10.1038/nn.3418

[b26] Endesfelder U., Malkusch S., Fricke F. & Heilemann M. A simple method to estimate the average localization precision of a single-molecule localization microscopy experiment. Histochem. Cell. Biol. 141, 629–638 (2014). 24522395 10.1007/s00418-014-1192-3

[b27] Jiao W., Masich S., Franzén O. & Shupliakov O. Two pools of vesicles associated with the presynaptic cytosolic projection in *Drosophila* neuromuscular junctions. J. Struct. Biol. 172, 389–394 (2010). 20678577 10.1016/j.jsb.2010.07.007

[b28] Atwood H. L., Govind C. K. & Wu C.-F. Differential ultrastructure of synaptic terminals on ventral longitudinal abdominal muscles in *Drosophila* larvae. J. Neurobiol. 24, 1008–1024 (1993). 8409966 10.1002/neu.480240803

[b29] Feeney C. J., Karunanithi S., Pearce J., Govind C. K. & Atwood H. L. Motor nerve terminals on abdominal muscles in larval flesh flies, *Sarcophaga bullata*: comparisons with *Drosophila*. J. Comp. Neurol. 402, 197–209 (1998). 9845243

[b30] Bar-On D. . Super-resolution imaging reveals the internal architecture of nano-sized syntaxin clusters. J. Biol. Chem. 287, 27158–27167 (2012). 22700970 10.1074/jbc.M112.353250 PMC3411058

[b31] Kim S. & Coulombe P. A. Intermediate filament scaffolds fulfill mechanical, organizational, and signaling functions in the cytoplasm. Genes Dev. 21, 1581–1597 (2007). 17606637 10.1101/gad.1552107

[b32] Patterson G., Davidson M., Manley S. & Lippincott-Schwartz J. Superresolution imaging using single-molecule localization. Annu. Rev. Phys. Chem. 61, 345–367 (2010). 20055680 10.1146/annurev.physchem.012809.103444 PMC3658623

[b33] Gilestro G. F., Tononi G. & Cirelli C. Widespread changes in synaptic markers as a function of sleep and wakefulness in *Drosophila*. Science 324, 109–112 (2009). 19342593 10.1126/science.1166673 PMC2715914

[b34] Graf E. R., Daniels R. W., Burgess R. W., Schwarz T. L. & Diantonio A. Rab3 dynamically controls protein composition at active zones. Neuron 64, 663–677 (2009). 20005823 10.1016/j.neuron.2009.11.002 PMC2796257

[b35] Peled E. S. & Isacoff E. Y. Optical quantal analysis of synaptic transmission in wild-type and *rab3*-mutant *Drosophila* motor axons. Nat. Neurosci. 14, 519–526 (2011). 21378971 10.1038/nn.2767 PMC7645962

[b36] Heckmann M. & Dudel J. Desensitization and resensitization kinetics of glutamate receptor channels from *Drosophila* larval muscle. Biophys. J. 72, 2160–2169 (1997). 9129818 10.1016/S0006-3495(97)78859-3 PMC1184410

[b37] Forsythe I. D., Tsujimoto T., Barnes-Davies M., Cuttle M. F. & Takahashi T. Inactivation of presynaptic calcium current contributes to synaptic depression at a fast central synapse. Neuron 20, 797–807 (1998). 9581770 10.1016/s0896-6273(00)81017-x

[b38] Hosoi N., Holt M. & Sakaba T. Calcium dependence of exo- and endocytotic coupling at a glutamatergic synapse. Neuron 63, 216–229 (2009). 19640480 10.1016/j.neuron.2009.06.010

[b39] Guerrero G. . Heterogeneity in synaptic transmission along a *Drosophila* larval motor axon. Nat. Neurosci. 8, 1188–1196 (2005). 16116446 10.1038/nn1526 PMC1402256

[b40] Holderith N. . Release probability of hippocampal glutamatergic terminals scales with the size of the active zone. Nat. Neurosci. 15, 988–997 (2012). 22683683 10.1038/nn.3137 PMC3386897

[b41] Matkovic T. . The Bruchpilot cytomatrix determines the size of the readily releasable pool of synaptic vesicles. J. Cell Biol. 202, 667–683 (2013). 23960145 10.1083/jcb.201301072 PMC3747298

[b42] Miskiewicz K. . ELP3 controls active zone morphology by acetylating the ELKS family member Bruchpilot. Neuron 72, 776–788 (2011). 22153374 10.1016/j.neuron.2011.10.010

[b43] Kurdyak P., Atwood H., Stewart B. & Wu C.-F. Differential physiology and morphology of motor axons to ventral longitudinal muscles in larval *Drosophila*. J. Comp. Neuro. 350, 463–472 (1994). 10.1002/cne.903500310 7884051

[b44] Karunanithi S., Marin L., Wong K. & Atwood H. Quantal size and variation determined by vesicle size in normal and mutant *Drosophila* glutamatergic synapses. J. Neurosci. 22, 10267 (2002). 12451127 10.1523/JNEUROSCI.22-23-10267.2002 PMC6758758

[b45] Hallermann S. . Bassoon speeds vesicle reloading at a central excitatory synapse. Neuron 68, 710–723 (2010). 21092860 10.1016/j.neuron.2010.10.026 PMC3004039

[b46] Zhang Q., Li Y. & Tsien R. W. The dynamic control of kiss-and-run and vesicular reuse probed with single nanoparticles. Science 323, 1448–1453 (2009). 19213879 10.1126/science.1167373 PMC2696197

[b47] Neher E. What is rate-limiting during sustained synaptic activity: vesicle supply or the availability of release sites. Front. Syn. Neurosci. 2, 1–6 (2010). 10.3389/fnsyn.2010.00144 PMC3059671 21423530

[b48] Endesfelder U. . Multiscale spatial organization of RNA polymerase in *Escherichia coli*. Biophys. J. 105, 172–181 (2013). 23823236 10.1016/j.bpj.2013.05.048 PMC3699759

[b49] Durisic N., Laparra-Cuervo L., Sandoval-Álvarez A., Borbely J. S. & Lakadamyali M. Single-molecule evaluation of fluorescent protein photoactivation efficiency using an in vivo nanotemplate. Nat. Methods 11, 156–162 (2014). 24390439 10.1038/nmeth.2784

[b50] Sugiyama Y., Kawabata I., Sobue K. & Okabe S. Determination of absolute protein numbers in single synapses by a GFP-based calibration technique. Nat. Methods 2, 677–684 (2005). 16118638 10.1038/nmeth783

[b51] Ulbrich M. H. & Isacoff E. Y. Subunit counting in membrane-bound proteins. Nat. Methods 4, 319–321 (2007). 17369835 10.1038/NMETH1024 PMC2744285

[b52] Endesfelder U. . Chemically induced photoswitching of fluorescent probes--a general concept for super-resolution microscopy. Molecules 16, 3106–3118 (2011). 21490558 10.3390/molecules16043106 PMC6260607

[b53] Takamori S. . Molecular anatomy of a trafficking organelle. Cell 127, 831–846 (2006). 17110340 10.1016/j.cell.2006.10.030

[b54] Boyken J. . Molecular profiling of synaptic vesicle docking sites reveals novel proteins but few differences between glutamatergic and GABAergic synapses. Neuron 78, 285–297 (2013). 23622064 10.1016/j.neuron.2013.02.027

[b55] Sieber J. J. . Anatomy and dynamics of a supramolecular membrane protein cluster. Science 317, 1072–1076 (2007). 17717182 10.1126/science.1141727

[b56] Stewart B. A., Atwood H. L., Renger J. J., Wang J. & Wu C.-F. Improved stability of *Drosophila* larval neuromuscular preparations in haemolymph-like physiological solutions. J. Comp. Physiol. A 175, 179–191 (1994). 8071894 10.1007/BF00215114

[b57] Wu Y., Kawasaki F. & Ordway R. W. Properties of short-term synaptic depression at larval neuromuscular synapses in wild-type and temperature-sensitive paralytic mutants of *Drosophila*. J. Neurophysiol. 93, 2396–2405 (2005). 15845998 10.1152/jn.01108.2004

[b58] Trommershäuser J., Schneggenburger R., Zippelius A. & Neher E. Heterogeneous presynaptic release probabilities: functional relevance for short-term plasticity. Biophys. J. 84, 1563–1579 (2003). 12609861 10.1016/S0006-3495(03)74967-4 PMC1302728

[b59] Schäfer P., van de Linde S., Lehmann J., Sauer M. & Doose S. Methylene blue- and thiol-based oxygen depletion for super-resolution imaging. Anal. Chem. 85, 3393–3400 (2013). 23410003 10.1021/ac400035k

[b60] Sharonov A. & Hochstrasser R. M. Single-molecule imaging of the association of the cell-penetrating peptide Pep-1 to model membranes. Biochemistry 46, 7963–7972 (2007). 17567046 10.1021/bi700505h

[b61] Tokunaga M., Imamoto N. & Sakata-Sogawa K. Highly inclined thin illumination enables clear single-molecule imaging in cells. Nat. Methods 5, 159–161 (2008). 18176568 10.1038/nmeth1171

[b62] Wolter S. . Real-time computation of subdiffraction-resolution fluorescence images. J. Microsc. 237, 12–22 (2010). 20055915 10.1111/j.1365-2818.2009.03287.x

[b63] Wolter S. . rapidSTORM: accurate, fast open-source software for localization microscopy. Nat. Methods 9, 1040–1041 (2012). 23132113 10.1038/nmeth.2224

[b64] Wolter S., Endesfelder U., van de Linde S., Heilemann M. & Sauer M. Measuring localization performance of super-resolution algorithms on very active samples. Opt. Express 19, 7020–7033 (2011). 21503016 10.1364/OE.19.007020

[b65] Ester M., Kriegel H.-P. & Sander J. in Proc. 2nd Internat. Conf. on Knowledge Discovery and Data Mining (KDD-96) 226–231München, Germany (1996).

