# Supplementary material for: Quantitative super-resolution imaging of Bruchpilot distinguishes active zone states
Source: Nat Commun. 2014 Aug 18;5:4650. doi: 10.1038/ncomms5650 (PMC4143948; doi:10.1038/ncomms5650)
Supplement: Supplementary Figures, Table and References — Supplementary Figures 1-6, Supplementary Table 1 and Supplementary References [file ncomms5650-s1.pdf]

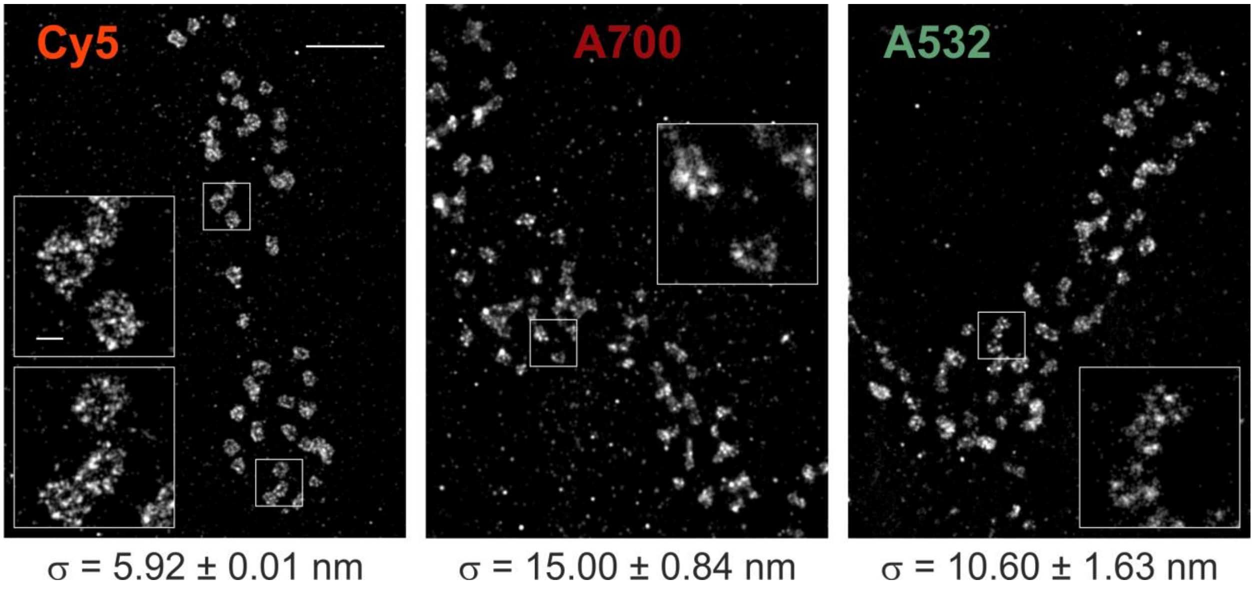

Supplementary Figure 1. Super-resolution imaging of wild type CAZs with three different photoswitchable fluorophores under *d*STORM conditions. Using Cy5, Alexa Fluor 700 (A700), and Alexa Fluor 532 (A532) as photoswitchable fluorophores, individual wt CAZ units can be resolved with *d*STORM. However, the CAZ ultrastructure is most accurately resolved by Cy5 (see insets). This is also demonstrated by the localization precision (σ) within the CAZ, which was determined according to Endesfelder *et al*.[1]. This comparison suggested the use of Cy5 over A532 and A700. Scale bars 2 µm and 200 nm (inset).

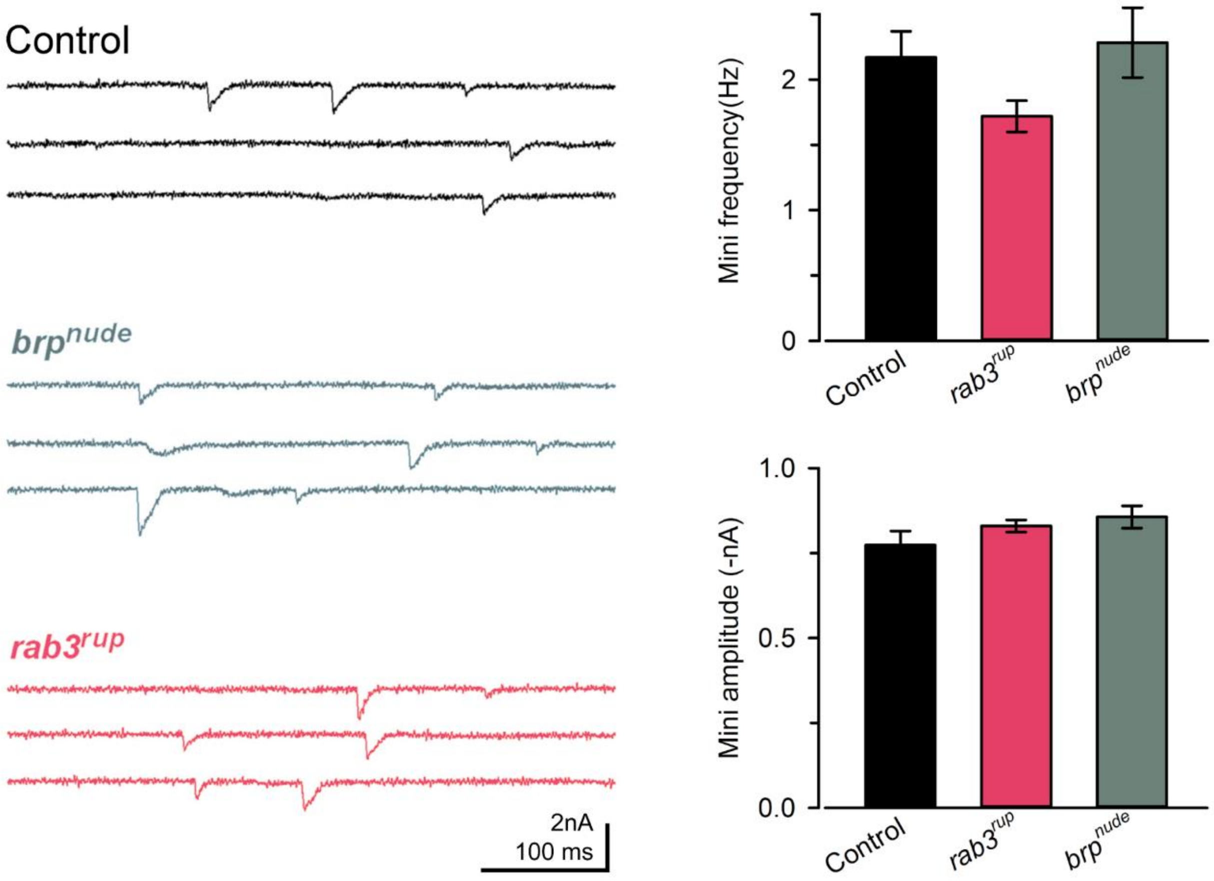

Supplementary Figure 2. **Similar spontaneous transmitter release at larval NMJs.** Example traces and quantification of data (mean ± SEM). Both the frequency (Control: 2.17 ± 0.20 Hz SEM, n = 11 NMJs; *brp*^{nude}: 2.28 ± 0.27 Hz, n = 17, rank sum test P = 0.96 vs. Control; *rab3*^{rup}: 1.72 ± 0.12 Hz, n = 17, rank sum test P = 0.07 vs. Control) and the amplitude (Control: -0.77 ± 0.04 nA SEM, n = 11 NMJs; *brp*^{nude}: -0.86 ± 0.03 nA, n = 17, rank sum test P = 0.20 vs. Control; *rab3*^{rup}: -0.83 ± 0.02 nA, n = 17, rank sum test P = 0.26 vs. Control) of *rab3*^{rup} and *brp*^{nude} minis were similar to those of controls.

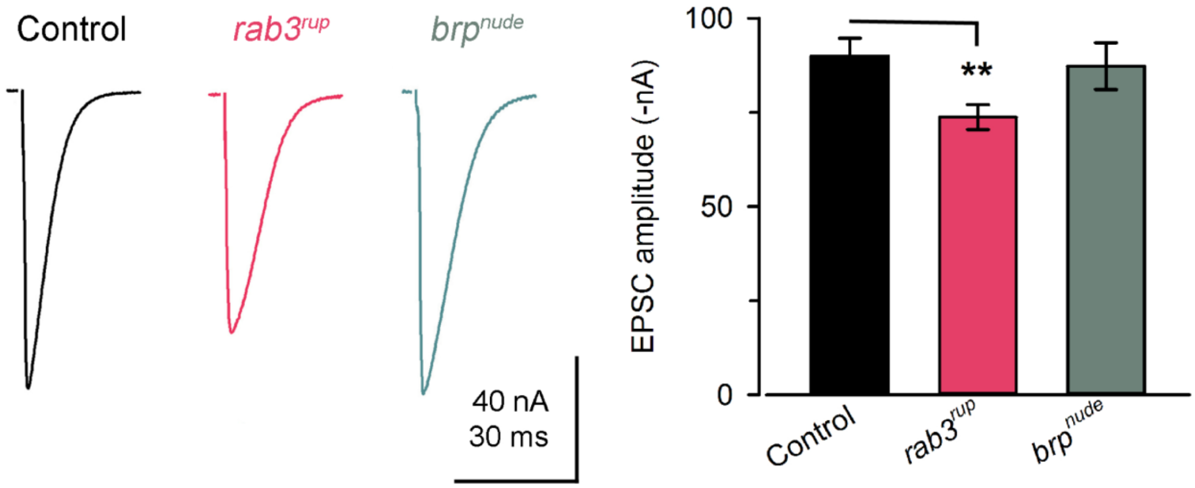

Supplementary Figure 3. Only subtle differences in basal evoked transmitter release. Average traces during low-frequency stimulation (0.2 Hz) and summary of data (mean ± SEM). The average eEPSC amplitude at *rab3*^{rup} NMJs was slightly below wild-type levels (Control: -89.9 ± 4.8 nA SEM, n = 21 NMJs; *brp*^{nude}: -87.3 ± 6.2 nA, n = 26, rank sum test P = 0.45 vs. Control; *rab3*^{rup}: -73.8 ± 3.3 nA, n = 28, rank sum test P = 0.009 vs. Control).

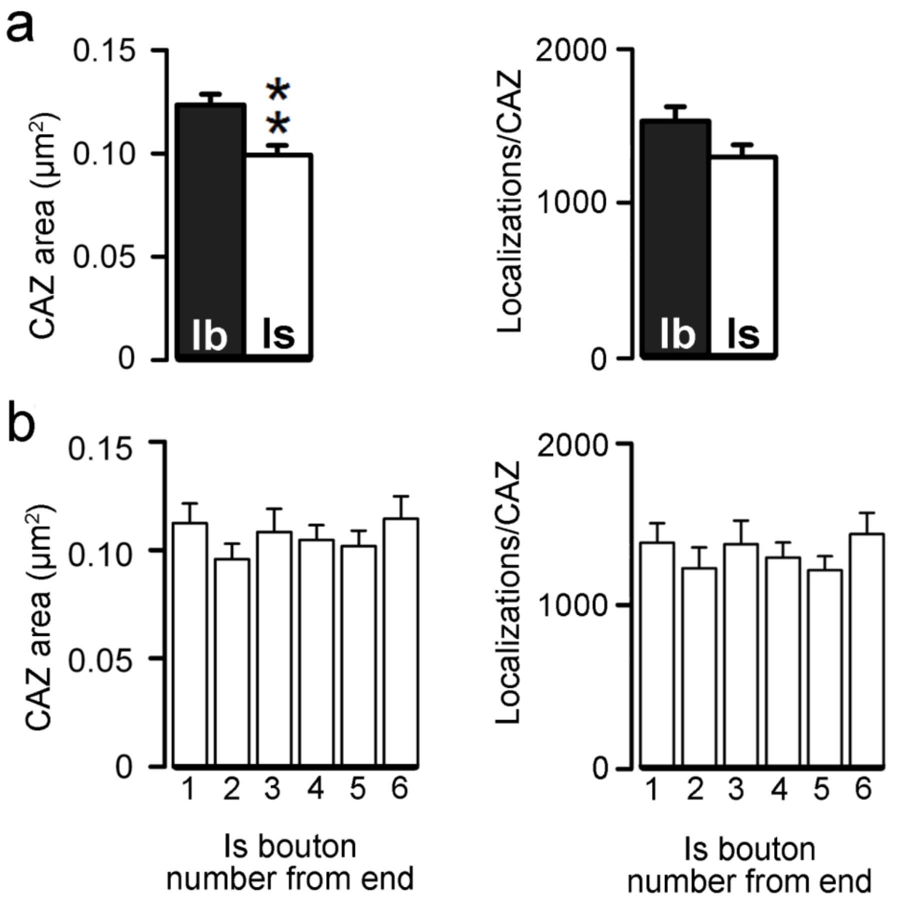

Supplementary Figure 4. CAZ ultrastructure of type Is motorneurons. (**a**) Ultrastructural analysis shows that the average CAZ of type Is boutons is smaller than Ib (Ib: 0.1235 ± 0.005 μm^2 SEM, n = 20 NMJs, 1,255 CAZs; Is: 0.0992 ± 0.005 μm^2, n = 21 NMJs, 1,042 CAZs, rank sum test P = 0.002), while the number of Brp localizations does not differ significantly (Ib: 1,510 ± 93 SEM, n = 20 NMJs; Is: 1,282 ± 78, n = 21 NMJs, rank sum test P = 0.074). (**b**) In contrast to type Ib CAZs (Fig. 6), type-Is CAZs displayed no gradient along the motorneuron (CAZ area: 0.1121 ± 0.009 SEM (distal), n = 108, 0.1141 ± 0.01 (proximal), n = 77, rank sum test P = 0.575; Localizations/CAZ: 1,380 ± 120 SEM (distal), n= 108, 1,433 ± 130 (proximal), n = 77, rank sum test P = 0.445). Data are presented as mean ± SEM.

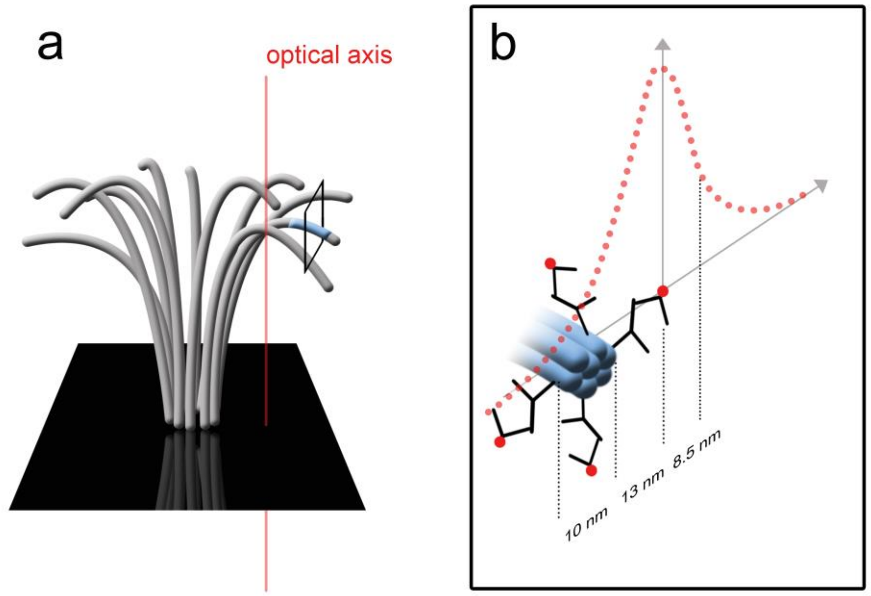

Supplementary Figure 5. Organization of fluorophores around Brp filaments in space. (**a**) Cartoon of a CAZ-unit with approximate region of the mAb Brp^{Nc82} epitope shaded blue. For the cluster analysis of Brp localizations, CAZ-units were viewed *en face*, i.e. with the presynaptic membrane perpendicular to the optical axis (indicated by red line). (**b**) Slice through a CAZ filament at the level of the Nc82 epitope (boxed region in a). Graphical scheme illustrating the orientation of antibody complexes (mAb Brp^{Nc82} and Cy5-labelled [red] $F(ab')_2$ fragment) around Brp epitopes (blue) aligned along the protein's long axis. Seven Brp proteins are suggested to contribute to one EM filament on average. For the sake of clarity, only four Ab complexes (each labelled with one fluorophore) are shown. Taking into account the dimensions of the Ab complex (~13 nm), the *d*STORM localization precision (6-7 nm standard deviation, ~17 nm FWHM; Fig. 1a,b; Gaussian fit indicated by dotted red line for one Cy5), and the EM filament diameter (~10 nm; Jiao et al., 2010), localizations may be separated by roughly 53 nm. Multiprotein filaments may bend outwards from the CAZ-unit centre[2,3]. At the level of the Nc82 epitope, the filament's long axis will be perpendicular to the optical axis, and the separation of localizations along the z-axis will be reduced to roughly 43 nm in x and y (the 10 nm filament diameter does not contribute). In good agreement with this calculation, the elliptical clusters are on average about 60 nm (long axis diameter) $\times$ 40 nm (short axis diameter) in size.

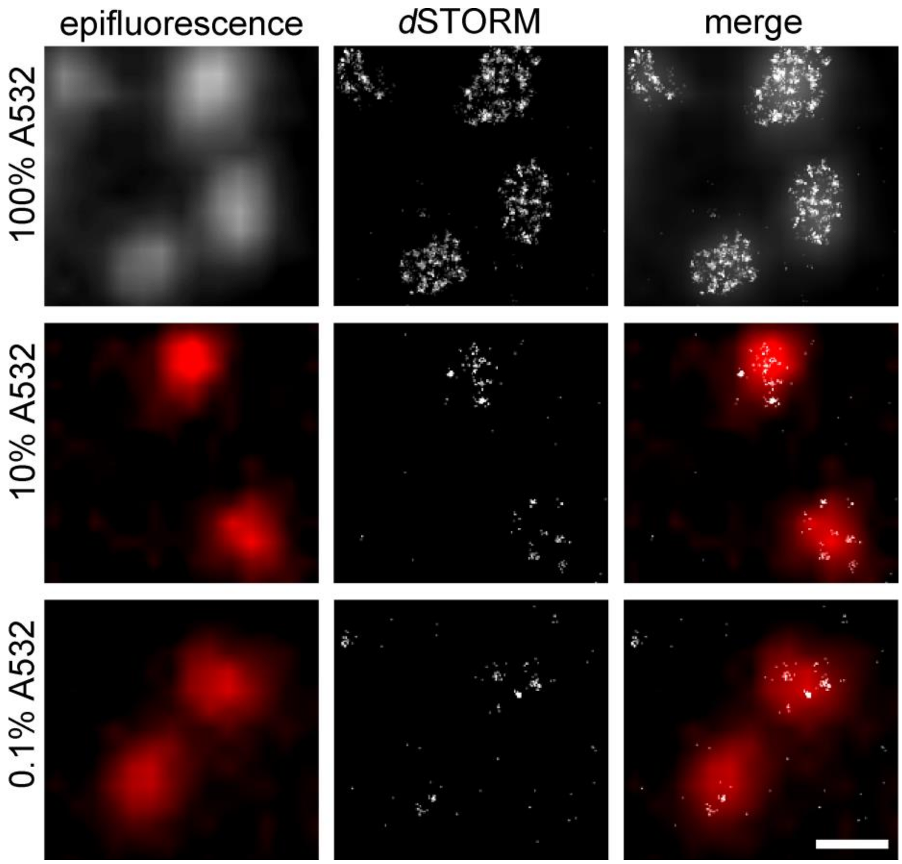

Supplementary Figure 6. Titration of A532-labelled secondary antibodies. Experiments were performed with primary mAb Brp^{Nc82} (1/2,000) and dilutions of A532-labelled secondary Ab (grey; 1, 1/10, 1/1,000). Brp was co-stained with A700 (red) to give an overall constant secondary Ab concentration ($6.25x10^{-9}$ M). The epifluorescence signal was used to identify the CAZ at low concentrations of A532. Shown are examples at different A532-labelled secondary Ab concentrations. For *d*STORM images a sub-pixel binning of 10 nm/px was applied. Scale bar: 500 nm.

Genotype	N		p		k_{+1}	
	model 1	model 2	model 1	model 2	model 1	model 2
Control, n = 10	555 ± 57	622 ± 76	0.22 ± 0.02	0.23 ± 0.04	0.61 ± 0.05	0.68 ± 0.1
brp^{nude}, n = 10	612 ± 64	663 ± 68	0.2 ± 0.01	0.19 ± 0.01	0.42 ± 0.04	0.42 ± 0.03
rab3^{rup}, n = 11	246 ± 27	263 ±30	0.54 ± 0.09	0.53 ± 0.09	0.54 ± 0.05	0.58 ± 0.07

Supplementary Table 1. Modelling parameters. Data are presented as mean ± SEM, n indicates number of individually fitted experiments. Kruskal-Wallis tests with Dunn's Multiple Comparison tests indicate the following levels of significance:

$N_{model\ 1}$ P = 0.0002, Control vs. *brp*^{nude} P > 0.05, Control vs. *rab3*^{rup} P < 0.001

$N_{model\ 2}$ P = 0.0001, Control vs. *brp*^{nude} P > 0.05, Control vs. *rab3*^{rup} P < 0.001

$p_{model\ 1}$ P = 0.002, Control vs. *brp*^{nude} P > 0.05, Control vs. *rab3*^{rup} P < 0.01

$p_{model\ 2}$ P = 0.002, Control vs. *brp*^{nude} P > 0.05, Control vs. *rab3*^{rup} P < 0.01

$k_{+1,\ model\ 1}$ P = 0.005, Control vs. *brp*^{nude} P < 0.01, Control vs. *rab3*^{rup} P > 0.05

$k_{+1,\ model\ 2}$ P = 0.006, Control vs. *brp*^{nude} P < 0.01, Control vs. *rab3*^{rup} P > 0.05

Supplementary references

1. Endesfelder, U., Malkusch, S., Fricke, F. & Heilemann, M. A simple method to estimate the average localization precision of a single-molecule localization microscopy experiment. *Histochem Cell Biol* (2014) doi:10.1007/s00418-014-1192-3.
2. Jiao, W., Masich, S., Franzén, O. & Shupliakov, O. Two pools of vesicles associated with the presynaptic cytosolic projection in *Drosophila* neuromuscular junctions. *J Struct Biol* **172**, 389–394 (2010).
3. Wichmann, C. & Sigrist, S. The active zone T-bar—a plasticity module? *J Neurogenet* (2010).
